# Binocular fusion and invariant category learning due to predictive remapping during scanning of a depthful scene with eye movements

**DOI:** 10.3389/fpsyg.2014.01457

**Published:** 2015-01-14

**Authors:** Stephen Grossberg, Karthik Srinivasan, Arash Yazdanbakhsh

**Affiliations:** Center for Adaptive Systems, Graduate Program in Cognitive and Neural Systems, Center of Excellence for Learning in Education, Science and Technology, Center for Computational Neuroscience and Neural Technology, and Department of MathematicsBoston University, Boston, MA, USA

**Keywords:** depth perception, perceptual stability, predictive remapping, saccadic eye movements, object recognition, spatial attention, gain fields, category learning

## Abstract

How does the brain maintain stable fusion of 3D scenes when the eyes move? Every eye movement causes each retinal position to process a different set of scenic features, and thus the brain needs to binocularly fuse new combinations of features at each position after an eye movement. Despite these breaks in retinotopic fusion due to each movement, previously fused representations of a scene in depth often appear stable. The 3D ARTSCAN neural model proposes how the brain does this by unifying concepts about how multiple cortical areas in the What and Where cortical streams interact to coordinate processes of 3D boundary and surface perception, spatial attention, invariant object category learning, predictive remapping, eye movement control, and learned coordinate transformations. The model explains data from single neuron and psychophysical studies of covert visual attention shifts prior to eye movements. The model further clarifies how perceptual, attentional, and cognitive interactions among multiple brain regions (LGN, V1, V2, V3A, V4, MT, MST, PPC, LIP, ITp, ITa, SC) may accomplish predictive remapping as part of the process whereby view-invariant object categories are learned. These results build upon earlier neural models of 3D vision and figure-ground separation and the learning of invariant object categories as the eyes freely scan a scene. A key process concerns how an object's surface representation generates a form-fitting distribution of spatial attention, or attentional shroud, in parietal cortex that helps maintain the stability of multiple perceptual and cognitive processes. Predictive eye movement signals maintain the stability of the shroud, as well as of binocularly fused perceptual boundaries and surface representations.

## 1. Introduction

### 1.1. Stability of 3D percepts across eye movements

Our eyes continually move from place to place as they scan a scene to fixate different objects with their high resolution foveal representations. Despite the evanescent nature of each fixation, we perceive the world continuously in depth. Such percepts require explanation, if only because each eye movement causes the fovea to process a different set of scenic features, and thus there are breaks in retinotopic fusion due to each movement. Within a considerable range of distances and directions of movement, the fused scene appears stable in depth, despite the fact that new retinotopic matches occur after each movement. How does the brain convert such intermittent fusions into a stable 3D percept that persists across eye movements?

This article develops the 3D ARTSCAN model to explain and simulate how the brain does this, and makes several predictions to further test model properties. The model builds upon and integrates concepts and mechanisms from earlier models:

FACADE (Form-And-Color-And-DEpth) is a theory of 3D vision and figure-ground separation that proposes how 3D boundaries and surfaces are formed from 3D scenes and 2D pictures that may include partially occluding objects (Grossberg, 1994, 1997; Grossberg and McLoughlin, 1997; Grossberg and Kelly, 1999; Kelly and Grossberg, 2000; Grossberg et al., 2002, 2007, 2008; Grossberg and Swaminathan, 2004; Cao and Grossberg, 2005, 2012; Grossberg and Yazdanbakhsh, 2005; Fang and Grossberg, 2009). The articles that develop FACADE also summarize and simulate perceptual and neurobiological data supporting the model's prediction that 3D boundary and surface representations are, indeed, the perceptual units of 3D vision.

aFILM (Anchored Filling-In Lightness Model) simulates psychophysical data about how the brain generates representations of anchored lightness and color in response to psychophysical displays and natural scenes (Hong and Grossberg, 2004; Grossberg and Hong, 2006).

ARTSCAN (Grossberg, 2007, 2009; Fazl et al., 2009) models and simulates perceptual, attentional, and neurobiological data about how the brain can coordinate spatial and object attention across the Where and What cortical streams to learn and recognize view-invariant object category representations as it scans a 2D scene with eye movements. These category representations form in the inferotemporal cortex in response to 2D boundary and surface representations that form across several parts of the visual cortex. In order to learn view-invariant object categories, the model showed how spatial attention maintains its stability in head-centered coordinates during eye movements as a result of the action of eye-position-sensitive gain fields.

These earlier models did not, however, consider how 3D boundary and surface representations that are formed from binocularly fused information from the two eyes is maintained as the eyes move to fixate different sets of object features. The current article shows how the stability of 3D boundary and surface representations *and* of spatial attention are ensured using gain fields. With this new competence incorporated, the 3D ARTSCAN model can learn view-invariant object representations as the eyes scan a depthful scene.

3D ARTSCAN is also consistent with the pARTSCAN (positional ARTSCAN) model (Cao et al., 2011), which clarifies how an observer can learn both positionally-invariant and view-invariant object categories in a 2D scene; the dARTSCAN (distributed ARTSCAN) model (Foley et al., 2012), which clarifies how visual backgrounds do not become dark when spatial attention is focused on a particular object, how Where stream transient attentional components and What stream sustained attentional components interact, and how prefrontal priming interacts with parietal attention mechanisms to influence search efficiency; and the ARTSCAN Search model (Chang et al., 2014), which, in addition to supporting view- and positionally-invariant object category learning and recognition using Where-to-What stream interactions, can also search a scene for a valued goal object using reinforcement learning, cognitive-emotional interactions, and What-to-Where stream interactions. It thereby proposes a neurobiologically-grounded solution of the Where's Waldo problem. With the capacity of searching objects in depth added, which the results hereby about 3D perceptual stability permit, a 3D ARTSCAN Search model could learn and recognize both positionally-invariant and view-invariant object categories in a depthful scene, and use eye movements to search for a Where's Waldo target in such a scene, without disrupting perceptual stability during the search.

Section 1 summarizes conceptual issues and processes that are needed to understand and model the maintenance of 3D perceptual stability across saccadic eye movements. Section 2 heuristically reviews the ARTSCAN model upon which the 3D ARTSCAN model builds. Section 3 provides a heuristic description of 3D ARTSCAN concepts and mechanisms. Section 4 summarizes simulation results using the 3D ARTSCAN model that demonstrate 3D perceptual stability across saccadic eye movements. Section 5 summarizes the mathematical equations and parameters that define the 3D ARTSCAN model. Sections 3 and 5 are written with a parallel structure, and with cross-references to model equation numbers and model system diagrams, in order to facilitate model understanding. Section 6 provides a comparative discussion of key concepts and their relationships to other data and models. A reader can skip from Section 4 to 6 if the mathematical structure of the model is not of primary interest.

The main theoretical goal of the current article is to demonstrate the property of perceptual stability of 3D visual boundaries and surfaces across saccadic eye movements, which has been clarified using a variety of experimental paradigms (Irwin, 1991; Carlson-Radvansky, 1999; Cavanagh et al., 2001; Fecteau and Munoz, 2003; Henderson and Hollingworth, 2003; Beauvillain et al., 2005). The article also predicts how this process interacts with processes of spatial and object attention, invariant object category learning, predictive remapping, and eye movement control, notably how they all regulate and/or respond to adaptive coordinate transformations. As explained more fully below, the brain can prevent a break in binocular fusion after an eye movement occurs by using predictive gain fields to maintain 3D boundary and surface representations in head-centered coordinates, even though these representations are not maintained in retinotopic coordinates. This property is demonstrated by simulations using 2D geometrical shapes and natural objects that are viewed in 3D. In particular, the simulations show that the 3D boundary and surface representations of these objects are maintained in head-centered coordinates as the eyes move.

These simulation results generalize immediately to 3D objects that have multiple 2D planar surfaces, since the simulations due not depend upon a particular binocular disparity. Indeed, other modeling studies have demonstrated how the same retinotopic binocular mechanisms can process object features at multiple disparities (Grossberg and McLoughlin, 1997; Grossberg and Howe, 2003; Cao and Grossberg, 2005, 2012), including objects perceived from viewing stereograms (Fang and Grossberg, 2009) and natural 3D scenes (Cao and Grossberg, submitted), as well as objects that are slanted in depth (Grossberg and Swaminathan, 2004). All these results should be preserved under the action of predictive gain fields to convert their retinotopic boundary and surface representations into head-centered ones, since the gain fields merely predictively shift the representations that are created by the retinotopic mechanisms. The key point is thus that the gain field mechanism does not disrupt the retinotopically computed 3D boundary and surface representations. It just changes their coordinates from retinotopic to head-centered to create invariance under eye movements.

The current model computes target positions to which the eyes are commanded to move, but does not model the neural machinery that is needed to accomplish the yoked saccadic movements themselves. Earlier models of the saccadic and smooth pursuit eye movement brain systems that are commanded by such positional representations can be used to augment the current model in future studies (e.g., Grossberg and Kuperstein, 1986; Grossberg et al., 1997, 2012; Gancarz and Grossberg, 1998, 1999; Srihasam et al., 2009; Silver et al., 2011).

### 1.2. Predictive remapping and gain fields: maintaining fusion across saccades

The brain compensates for the changes in retinal coordinates of fused object features fast enough to prevent fusion from being broken. This compensatory property is called *predictive remapping*. Predictive remapping has been used to interpret neurophysiological data about the updating of the representation of visual space by intended eye movements, particularly in cortical areas such as the parietal cortex, prestriate cortical area V4, and frontal eye fields (Duhamel et al., 1992; Umeno and Goldberg, 1997; Gottlieb et al., 1998; Tolias et al., 2001; Sommer and Wurtz, 2006; Melcher, 2007, 2008, 2009; Saygin and Sereno, 2008; Mathot and Theeuwes, 2010a). Predictive remapping is often explained as being achieved by *gain fields* (Andersen and Mountcastle, 1983; Andersen et al., 1985; Grossberg and Kuperstein, 1986; Gancarz and Grossberg, 1999; Deneve and Pouget, 2003; Pouget et al., 2003), which enable featural representations to incorporate information about the current or predicted gaze position. Gain fields are populations of cells that enable movement-sensitive transformations to occur between one coordinate frame (say, retinotopic), whose representations change due to eye movements, and another (say, head-centered), whose representations are invariant under eye movements.

In both the ARTSCAN model and the 3D ARTSCAN model, gain fields are proposed to be updated by corollary discharges of outflow movement signals that act before the eyes stabilize on their next movement target. In the ARTSCAN model, these predictive gain field signals maintain the stability of spatial attention to an object as eye movements scan the object; see Section 2. In the 3D ARTSCAN model, gain field signals also prevent binocularly-fused object boundary and surface representations of the object from being reset by such eye movements. The 3D ARTSCAN model hereby proposes how the process of predictive remapping of 3D boundary and surface representations is linked to the processes of figure-ground separation of multiple objects in a scene, and of learning to categorize and attentively recognize these objects during active scanning of the scene with saccadic eye movements. The following sections summarize how these processes are predicted to be coordinated.

## 2. Review of ARTSCAN model

### 2.1. Solving the view-to-object binding problem while scanning a scene

The ARTSCAN model and its variants propose answers to the following basic questions: What is an object? How does the brain learn what an object is under both unsupervised and supervised learning conditions? ARTSCAN predicts how spatial and object attention are coordinated to achieve rapid object learning and recognition during eye movement search. In particular, ARTSCAN proposes how the brain learns to recognize an object when it is seen from multiple views, or perspectives. How does such view-invariant object category learning occur?

As the eyes scan a scene, two successive eye movements may focus on different parts of the same object or on different objects. ARTSCAN proposes how the brain avoids erroneously classifying views of different objects together, even before the brain knows what the object is. ARTSCAN also proposes how the brain controls eye movements that enable it to learn multiple view-specific categories and to associately link them with view-invariant object category representations.

**Figure 1:**
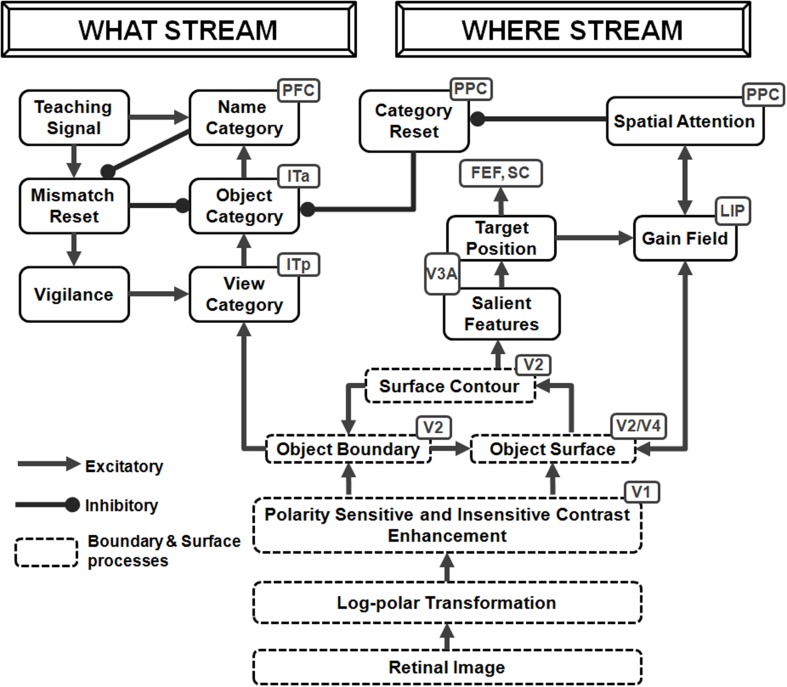
**Model diagram of the ARTSCAN model (reprinted with permission from Chang et al., 2014)**. A few simplified stages from the FACADE model (Grossberg and Todorović, 1988; Grossberg, 1994, 1997; Grossberg and McLoughlin, 1997) preprocess 2D images. The 3D ARTSCAN model is a synthesis and further development of the ARTSCAN model, the aFILM model of anchored lightness and color perception (Hong and Grossberg, 2004; Grossberg and Hong, 2006), and the FACADE model to enable 3D surface percepts to remain stable as saccadic eye movements scan a scene (as elaborated in Figures 2–**5**).

The ARTSCAN model (Figure 1) predicts how spatial attention may play a crucial role in controlling view-invariant object category learning, using attentionally-regulated signals from the Where cortical stream to the What cortical stream to modulate category learning. Several studies have reported that the distribution of spatial attention can configure itself to fit an object's form. Form-fitting spatial attention is sometimes called an *attentional shroud* (Tyler and Kontsevich, 1995). ARTSCAN explained how an object's pre-attentively formed surface representation in prestriate cortical area V4 may induce such a form-fitting attentional shroud in parietal cortex. In particular, feedback between the surface representation and the shroud are predicted to form a *surface-shroud resonance* that locks spatial attention on the object's surface. While this surface-shroud resonance remains active, it is predicted to accomplish the following: First, it ensures that eye movements tend to end at locations on the object's surface, thereby enabling different views of the same object to be sequentially explored (Theeuwes et al., 2010). Second, it keeps the emerging view-invariant object category active while different views of the object are learned by view-specific categories and associated with it.

The ARTSCAN model thus addressed what would otherwise appear to be an intractable infinite regress: If the brain does not already know what the object is, then how can it, without external guidance, prevent views from several objects from being associated and thus distort the learning of object categories? How does such unsupervised learning until naturalistic viewing conditions get started? The ARTSCAN model shows that an object's pre-attentively and automatically formed surface representation (Figure 1) provides the object-sensitive substrate that enables view-invariant object category learning to occur, and thereby circumvents this infinite regress.

The fact that a surface representation can form pre-attentively is consistent with the burgeoning psychophysical literature showing that 3D boundaries and surfaces are the units of pre-attentive visual perception (Grossberg and Mingolla, 1987; Grossberg, 1987a,b, 1994; Paradiso and Nakayama, 1991; Elder and Zucker, 1993; He and Nakayama, 1995; Rogers-Ramachandran and Ramachandran, 1998; Raizada and Grossberg, 2003) and that attention selects these units for recognition (Kahneman and Henik, 1981; He and Nakayama, 1995; LaBerge, 1995).

**Figure 2:**
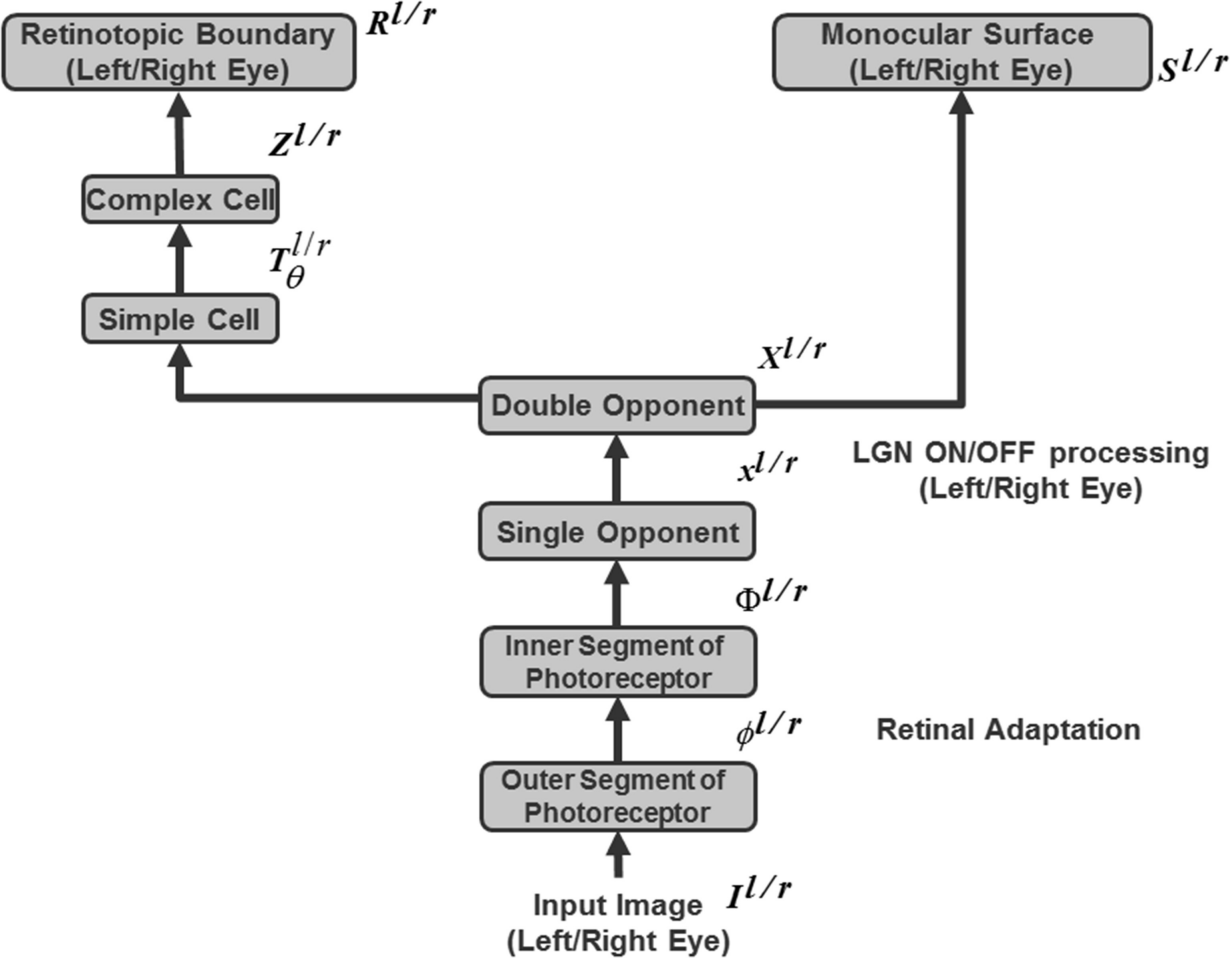
**Retinal adaptation of input scene followed by pre-attentive boundary and surface processing in the 3D ARTSCAN model**. Light adaptation at the model's outer segment of the photoreceptors and spatial contrast adaptation at the inner segments of photoreceptors are implemented as in the aFILM model (Grossberg and Hong, 2006) (Equations 1–8). The outputs from the inner segment of the photoreceptors input to the model LGN. These inputs are contrast-normalized by single opponent networks of ON and OFF cells via on-center off-surround and off-center on-surround interactions, respectively, among cells that obey membrane equation, or shunting, dynamics (Equations 9–14) and then by double-opponent networks (Equations 15, 16). LGN double-opponent outputs are used to compute orientationally- and contrast-selective simple cells that are selective to four different orientations (Equations 17–20). Simple cell outputs are pooled across all four orientations to yield complex cells (Equation 21). Complex cells, in turn, input to monocular left (L) and right (R) eye retinotopic boundaries.

**Figure 3:**
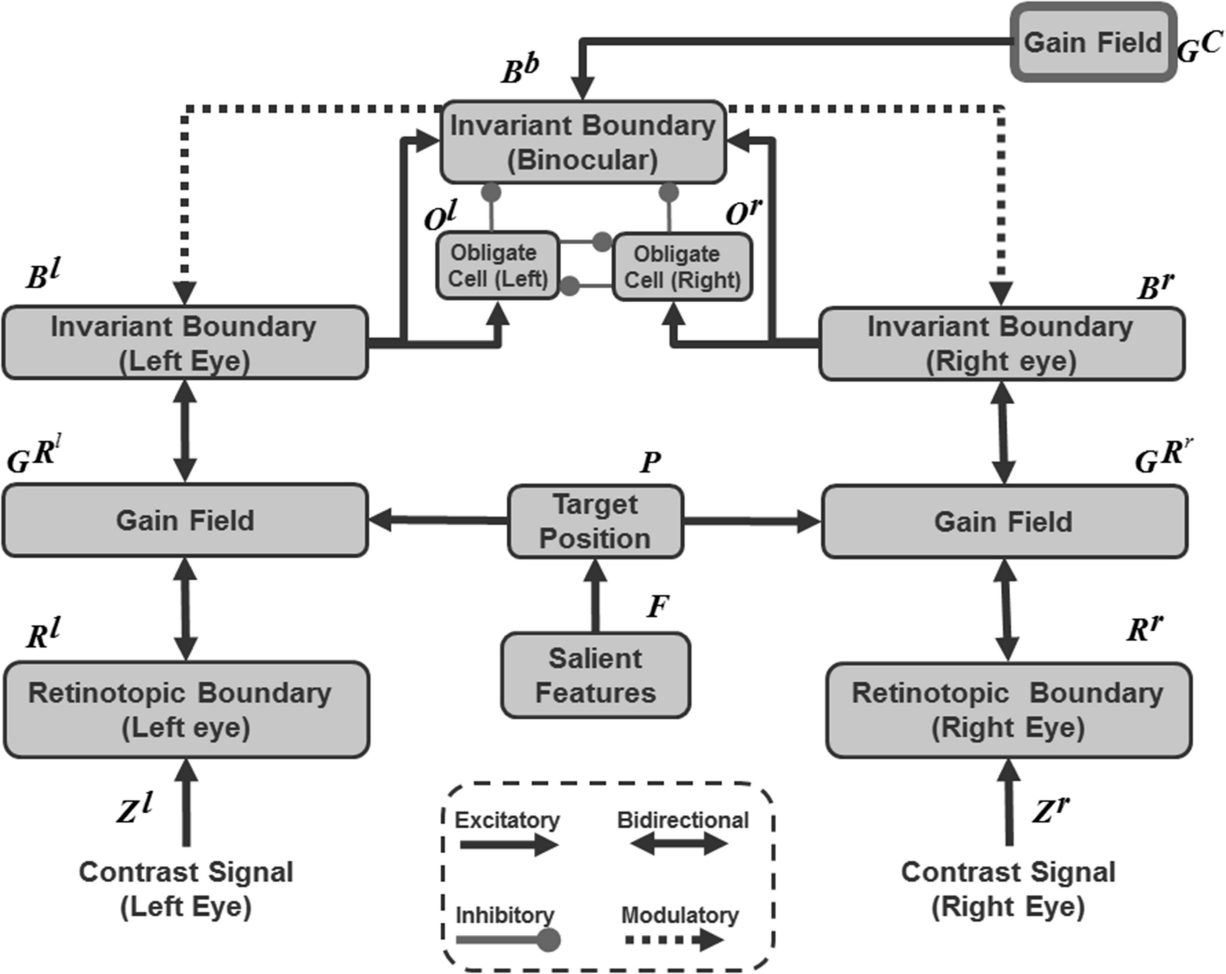
**3D ARTSCAN model macrocircuit for maintaining the stability of fused binocular boundaries during eye movements**. Retinotopic monocular boundaries (Equation 22) are computed from complex cell inputs (Equation 21). These boundaries are reset whenever the eyes move. The retinotopic monocular boundaries input to invariant monocular boundaries via gain fields. The invariant boundaries are not reset by eye movements because they are predictively remapped by eye position-selective gain fields before the eyes move to a new fixation position. The invariant monocular boundaries, in turn, feed back to modulate the retinotopic monocular boundaries. The gain fields receive their inputs from target positions that are computed from salient features on surface contours (see Sections 3.4, 3.6, and Equations 45, 64–66). The invariant monocular boundaries (Equation 26) are binocularly fused to form the invariant binocular boundaries (Equation 33). Both excitatory and inhibitory (obligate) inputs to the invariant binocular boundaries are needed to ensure their disparity selectivity. The maintained fusion of binocular boundaries is a primary goal of predictive remapping, since these boundaries support the persistence of object percepts during saccadic eye movements. These fused binocular boundaries modulate the activities of the invariant monocular boundaries and thus the activity of the retinotopic boundary layer via top-down feedback. This top-down feedback ensures that any changes or collapse in the invariant boundary activity is propagated all the way back to the retinotopic boundaries (see Section 3.3 and Equations 22–35).

**Figure 4:**
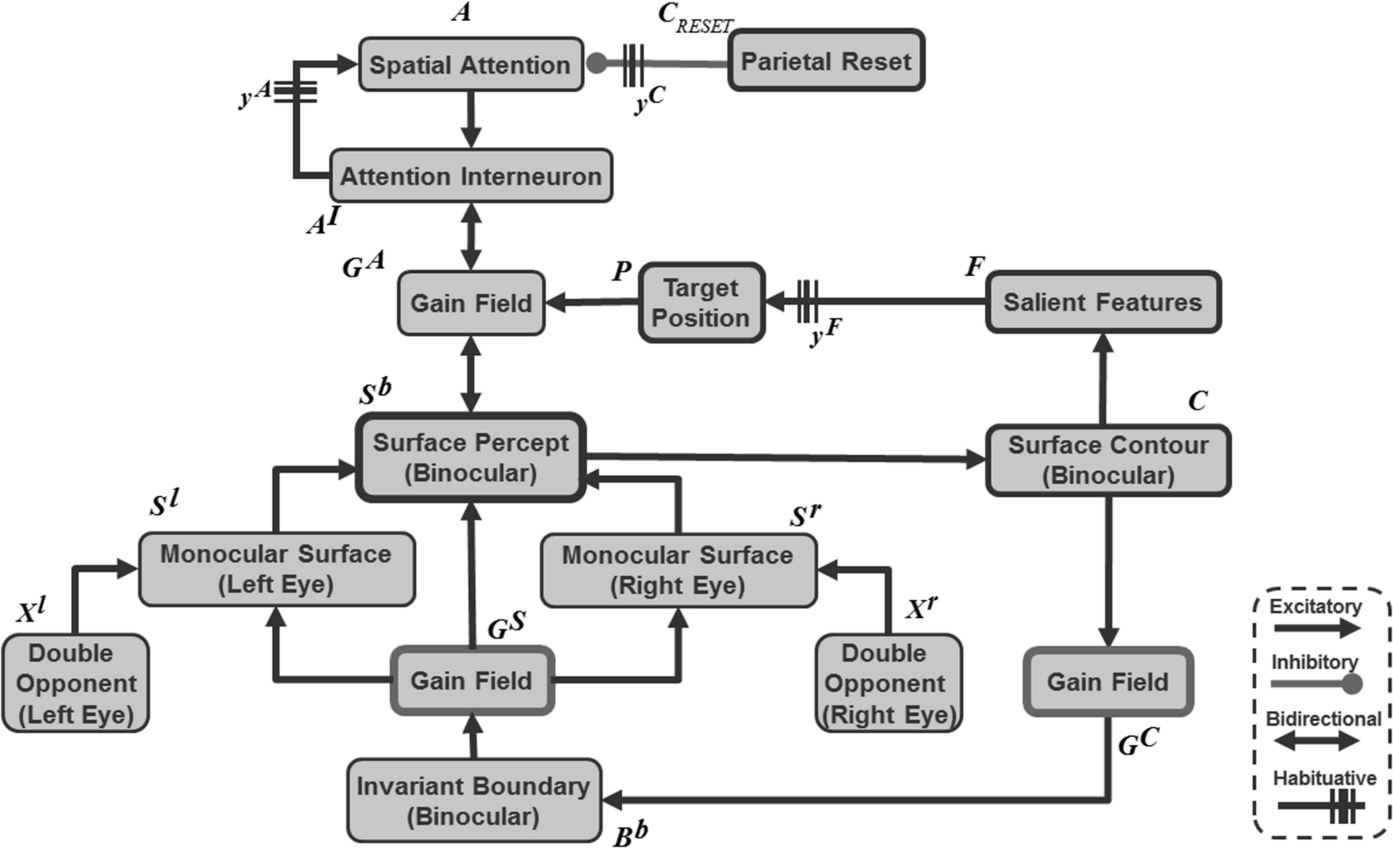
**Maintenance and perceptual stability of fused binocular surfaces during eye movements**. The monocular surfaces *S*^*l/r*^ fill-in (Equation 36) (Figure 2) brightness signals from the double-opponent ON and OFF cells (Equations 15, 16). The diffusion that governs filling-in is gated by invariant binocular boundaries (Equation 33) after they are converted into retinotopic binocular boundaries (Equation 40) via gain fields (Equations 42–44). The monocular surfaces are fused to form a binocular surface *S*^*b*^ (Equation 39). The rectified sum of the ON and OFF filling-in domains is the final binocular surface percept (Equation 41) and is assumed to be the consciously seen retinotopic surface percept in depth. Gain fields operating at different levels guarantee the stability of the binocular percept (Section 3.4 and Equations 36–50). Binocular surface representations give rise to surface contours *C* (Equation 45) from which the most salient feature positions *F* (Equation 64) are chosen as the next target positions *P* (Equation 66) for eye movements. Corollary discharges from the target positions are used to predictively remap key boundary and surface representations via gain fields (Section 3.6). In particular, a retinotopic binocular surface percept is remapped via gain fields (Equation 56) into attentional interneurons (Equation 55) that input to the spatial attention map at which a head-centered attentional shroud is chosen. The attentional shroud (Equation 51) habituates at an activity-dependent rate (Equation 61) and is inhibited by a burst of the parietal reset signal (Equation 62) that is rendered transient by its own habituative transmitter gate (Equation 63). This enables a shift in attention to occur to a different surface (see Sections 3.4–3.6 and Equations 36–66).

The ARTSCAN model used the simplest possible front end from the FACADE model of 3D vision and figure-ground perception (Grossberg, 1994, 1997; Grossberg and McLoughlin, 1997) in order to process letters of variable sizes and fonts in simple 2D images. The 3D ARTSCAN Search model elaborates this front end to enable binocular fusion of objects in a 3D scene (see Figures 2–**4** and Section 3 for details).

### 2.2. Attentional shroud inhibits reset of an invariant object category during object learning

ARTSCAN processes can be described as a temporally coordinated interaction between multiple brain regions within and between the What and Where cortical processing streams, including the Lateral Geniculate Nucleus (LGN), cortical areas V1, V2, V3A, V4, MT, MST, PPC, LIP, ITp, and ITa, and the superior colliculus (SC): The Where stream maintains an attentional shroud whose spatial coordinates mark the surface locations of a current “object of interest,” whose identity has yet to be determined in the What stream. As each view-specific category is learned by the What stream, say in posterior inferotemporal cortex (ITp), it focuses object attention via a learned top-down expectation on the critical features in the visual cortex (e.g., in prestriate cortical area V4) that will be used to recognize that view and its variations in the future. When the first such view-specific category is learned, it also activates a cell population at a higher cortical level, say anterior inferotemporal cortex (ITa), that will become the view-invariant object category.

Suppose that the eyes or the object move sufficiently to expose a new view whose critical features are significantly different from the critical features that are used to recognize the first view. Then the first view category is reset, or inhibited. This happens due to the mismatch of its learned top-down expectation, or prototype of attended critical features, with the newly incoming view information. This top-down prototype focuses object attention on the incoming visual information. Object attention hereby helps to control which view-specific categories are learned by determining when the currently active view-specific category should be reset, and a new view-specific category should be activated.

However, the view-invariant object category should *not* be reset every time a view-specific category is reset, or else it can never become view-invariant. This is what the attentional shroud accomplishes: It inhibits a tonically-active reset signal that would otherwise shut off the view-invariant category when each view-based category is reset. As the eyes foveate a sequence of views on a single object's surface through time, they trigger learning of a sequence of view-specific categories, and each of them is associatively linked through learning with the still-active view-invariant category.

When the eyes move off an object, its attentional shroud collapses in the Where stream, thereby transiently disinhibiting the reset mechanism that shuts off the view-invariant category in the What stream. When the eyes look at a different object, its shroud can form in the Where stream and a new view-specific category can be learned that can, in turn, activate the cells that will become a new view-invariant category in the What stream. Chiu and Yantis (2009) have described rapid event-related fMRI experiments in humans showing that a spatial attention shift causes a domain-independent transient parietal burst that correlates with a change of categorization rules. This transient parietal signal is a marker against which further experimental tests of model mechanisms can be based; e.g., a test of the predicted sequence of V4-parietal surface-shroud collapse (shift of spatial attention), transient parietal burst (reset signal), and collapse of currently active invariant object category in cortical area ITa (shift of categorization rules). These and related results (e.g., Corbetta et al., 2000; Yantis et al., 2002; Cabeza et al., 2008) are consistent with the model prediction of how different regions of the parietal cortex maintain sustained attention to a currently attended object (shroud) and control transient attention switching (reset burst) to a different object.

### 2.3. Boundary and surface representations form pre-attentively

Convergent psychophysical and neurobiological data (e.g., He and Nakayama, 1992; Elder and Zucker, 1998; Rogers-Ramachandran and Ramachandran, 1998; Lamme et al., 1999) support the 1984 prediction of Grossberg and colleagues that the units of pre-attentive visual perception are boundaries and surfaces (Cohen and Grossberg, 1984; Grossberg, 1984; Grossberg and Mingolla, 1985a,b; Grossberg and Todorović, 1988). The model that embodies this prediction is often called the BCS/FCS model, for Boundary Contour System and Feature Contour System. This hypothesis was generalized by Grossberg in 1987 to the prediction that 3D boundaries and surfaces are the units of 3D vision and figure-ground perception. This prediction is part of the FACADE (Form-And-Color-And-DEpth) theory of 3D vision and figure-ground separation, which has been used to explain and predict a wide range of perceptual and neurobiological data; see Grossberg (1994, 2003) and Raizada and Grossberg (2003) for reviews. Perceptual boundaries are predicted to form in the (LGN Parvo)-(V1 Interblob)-(V2 Interstripe)-V4 cortical stream, while perceptual surfaces are predicted to form in the (LGN Parvo)-(V1 Blob)-(V2 Thin Stripe)-V4 stream. Various psychophysical (Rubin, 1921; Beardslee and Wertheimer, 1958; Driver and Baylis, 1996), fMRI (Kourtzi and Kanwisher, 2001), and electrophysiological data (Baylis and Driver, 2001) support the hypothesis that boundaries and surfaces can form pre-attentively as they help to separate figures from their backgrounds in depth. These experiments show that whether an edge is assigned to a figure or to a background serves as an important factor for attracting attention, activating object recognition areas, and remembering it later. It has also been argued that, prior to attentive selection of an object, figure-ground segregation occurs (Baylis and Driver, 2001), and that it is yoked to bottom-up processes that do not need a top-down attentive influence to be initiated. The boundaries and surfaces that are implemented in the 3D ARTSCAN Search model are generalized in two ways beyond their implementation in the ARTSCAN model:

#### 2.3.1. 3D boundaries and surfaces

As noted above, the monocular boundaries and surfaces in the ARTSCAN model are generalized using FACADE theory mechanisms to form disparity-selective boundaries and surfaces that can represent an object in depth. In this generalization, processing stages for retinal adaptation as well as opponent and double-opponent processing in ON and OFF cells (Grossberg and Hong, 2006) feed into monocular and binocular laminar cortical boundary representations (Cao and Grossberg, 2005); see Sections 3 and 5 for details.

The surface representations that compete for spatial attention in shroud formation are called Filling-In Domains, or FIDOs (Grossberg, 1994). FACADE theory predicts that each of the depth-selective boundary representations that capture surface lightness and color at prescribed depths interacts with a complete set of opponent filling-in domains (light vs. dark, red vs. green, blue vs. yellow) that compete at each position. In addition, each FIDO's activity pattern is processed by an on-center off-surround shunting network that contrast-normalizes its input patterns (Grossberg, 1973, 1980). These two types of competition (opponent and spatial), acting together, define a double-opponent field of cells. There are multiple FIDOs, each sensitive to a different range of depths. These double-opponent FIDOs can represent conjunctions of depth and color across space. A unique conjunction of depth and color may pop out during visual search (Nakayama and Silverman, 1986) because it is the only active region on the FIDO corresponding to that depth and color. FACADE theory models its highest level of surface filling-in in cortical area V4, where visible surfaces are represented and 3D figure-ground separation is completed (e.g., Schiller and Lee, 1991).

These depth-selective double-opponent surface representations in V4 provide the computational substrates that compete for spatial attention in the model's parietal cortex. The reciprocal shroud-to-surface feedback may also be expected to be selective to conjunctions of depth and color. Such a mechanism may clarify various color-specific search data; e.g., Egeth et al. (1984) and Wolfe et al. (1994) wherein human subjects may break up a conjunctive search task into a color priming operation followed by depth-selective pop-out.

The 3D ARTSCAN Search model simulates a single depth-selective double-opponent FIDO, for simplicity.

#### 2.3.2. Predictive remapping maintains binocular fusion and shroud stability

In ARTSCAN, predictive remapping is used to maintain the stability of an attentional shroud as eye movements explore an attended object. This stability is needed to prevent the shroud from collapsing and disinhibiting the reset mechanism in response to every sufficiently large saccade that explores the object. In the current 3D ARTSCAN model, predictive remapping also has another role: it maintains binocular fusion of previously fused features as the eyes move within a certain spatial range to foveate a different set of features on the object. Thus, predictive remapping mechanisms that were previously predicted to operate in areas such as parietal cortex are here also suggested to operate as early as visual cortical area V1; see Sections 3.4, 3.5, and 5 for details.

The following sections summarize how the two types of predictive remapping are proposed to be related.

### 2.4. Surface contour signals initiate figure-ground separation

Shroud stability is achieved in ARTSCAN using feedback signals between surfaces and boundaries in the following way: 3D boundary signals are topographically projected from where they are formed in the V2 interstripes to the surface representations in the V2 thin stripes (Figure 1). These boundaries act both as *filling-in generators* that initiate the filling-in of surface lightness and color when the corresponding boundary and surface signals are aligned, and as *filling-in barriers* that prevent the filling-in of lightness and color from crossing object boundaries (Grossberg, 1994). If the boundary is closed, it can contain, or *gate*, the filling-in of an object's lightness and color within it. If, however, the boundary has a sufficiently big gap in it, then surface lightness and color can spread through the gap and surround the boundary on both sides, thereby equalizing the contrasts on both sides of the boundary.

Feedback from surfaces in V2 thin stripes to boundaries in V2 interstripes is achieved by *surface contour* signals. Surface contour signals are generated by contrast-sensitive on-center off-surround networks that generate contour-sensitive output signals from the activities across each FIDO after surface filling-in occurs. The inhibitory connections in the network's off-surround act across position and within depth. As a result, each FIDO generates output signals via its own contrast-sensitive on-center off-surround network. Surface contour signals are the output signals that are generated by contrast changes across each FIDO.

Such contrast changes typically occur if the filled-in surface is surrounded by gating signals from a closed boundary, because a closed boundary can contain a FIDO's filling-in process. In particular, gating at closed boundary positions generates contrasts of filled-in lightnesses or colors at these positions by blocking the spread of lightnesses or colors across these positions. As a result, surface contour signals can be generated at the positions where the gating signals of closed boundaries occur. The positions at which surface contour signals in the surface stream are generated are thus a subset of the same positions as those of the corresponding boundaries in the boundary stream. These boundary and surface contour positions typically include positions where there are salient features on an object's surface.

Surface contour signals are not, however, generated at boundary positions near a big gap, or hole, in an object boundary, since filled-in lightnesses and colors can flow out of, and around, such a boundary break to cause approximately equal filled-in activities on both sides of the boundary. Since there is then zero contrast of filled-in activity across such a boundary, the contrast-sensitive on-center off-surround network does not generate an output signal at these positions, and hence no surface contour forms there.

The boundary positions that limit the filling-in process within the surface stream are thus a superset of the positions in the surface stream at which surface contours form after filling-in. As a result, surface contour output signals back to the boundary stream are received at a subset of boundary positions. In particular, gating signals that are generated by closed boundaries block the flow of filled-in brightness and/or color signals outside the regions that they surround. Closed boundaries hereby mark the positions where a contrast different across space in the filled-in brightness and/or color can occur. They are therefore also positions where surface contour feedback signals can arise.

The surface contour feedback signals from the surface stream to the boundary stream are delivered via an on-center off-surround network that acts within position and across depth. The on-center signals strengthen the closed boundaries that generated the successfully filled-in surfaces, whereas the off-surround signals inhibit spurious boundaries at the same positions but farther depths. Surface contour signals hereby strengthen the boundaries that lead to successfully filled-in surfaces, while inhibiting those that do not. By eliminating spurious boundaries, the off-surround signals initiate figure-ground separation by enabling occluding and partially occluded surfaces to be separated onto different depth planes, and partially occluded boundaries and surfaces to be amodally completed behind their occluders. See Grossberg (1994), Kelly and Grossberg (2000), and Fang and Grossberg (2009) for further discussion of figure-ground percepts and computer simulations of them.

### 2.5. Attended surface contour signals create attention pointers to salient eye movement target positions

Figure-ground separation needs to occur at an earlier processing stage than the learning of view-specific and view-invariant categories of an object, since if different objects were not pre-attentively separated from each other, the brain would have no basis for segregating the learning of views that belong to one object. Once figure-ground separation is initiated, ARTSCAN predicts how surface contour signals can be used to determine a sequence of eye movement target positions to salient features on an attended object surface, and thus to enable multiple view-specific categories of the object to be learned and associated with an emerging view-invariant object category.

This works as follows: the pre-attentive bottom-up inputs from the retina and LGN activate multiple surface representations in cortical area V4. These surfaces, in turn, attempt to topographically activate spatial attention to form a surface-fitting attentional shroud in parietal cortex. As they do so, they generate top-down excitatory topographic feedback to visual cortex and long-range inhibitory interactions in parietal cortex. Taken together, these interactions define a *recurrent* on-center off-surround network that is capable of contrast-enhancing the strongest shroud and inhibiting weaker ones. Positive feedback from a winning shroud in parietal cortex to its surface in V4 is thus predicted to increase the contrast gain of the attended surface, as has been reported in both psychophysical experiments (Carrasco et al., 2000) and neurophysiological recordings from cortical areas V4 (Reynolds et al., 1999, 2000; Reynolds and Desimone, 2003), possibly carried by the known connections from parietal areas to V4 (Cavada and Goldman-Rakic, 1989, 1991; Distler et al., 1993; Webster et al., 1994).

How do salient features on an attended surface attract eye movements? If figure-ground separation begins in cortical area V2, with surface contours as one triggering mechanism, then these eye movement commands need to be generated no earlier than V2. The surface contour signals themselves are plausible candidates from which to derive eye movement target commands because, being generated by a contrast-sensitive on-center off-surround network, they are stronger at contour discontinuities and other distinctive contour features that are typical end points of saccadic movements. When the contrast of an attended surface increases, the strength of its surface contour signals also increases (Figure 1). Corollary discharges of these surface contour signals are predicted to be computed within a parallel pathway that is mediated via cortical area V3A (Nakamura and Colby, 2000; Caplovitz and Tse, 2007), which occurs after V2, and to generate saccadic commands that are restricted to salient features of the attended surface (Theeuwes et al., 2010) until the shroud collapses and spatial attention shifts to enshroud another object. Consistent with this prediction, it is known that “neurons within V3A···process continuously moving contour curvature as a trackable feature… not to solve the “ventral problem” of determining object shape but in order to solve the “dorsal problem” of what is going where” (Caplovitz and Tse, 2007, p. 1179).

In particular, ARTSCAN proposed how surface contour signals within the corollary discharge pathway are contrast-enhanced to select the largest signal as the next position upon which spatial attention will focus and the next saccadic eye movement will move (Figure 1). These positions have properties of the “attention pointers” reported by Cavanagh et al. (2010).

### 2.6. Predictive surface contour signals control gain fields that maintain shroud stability

**Figure 5:**
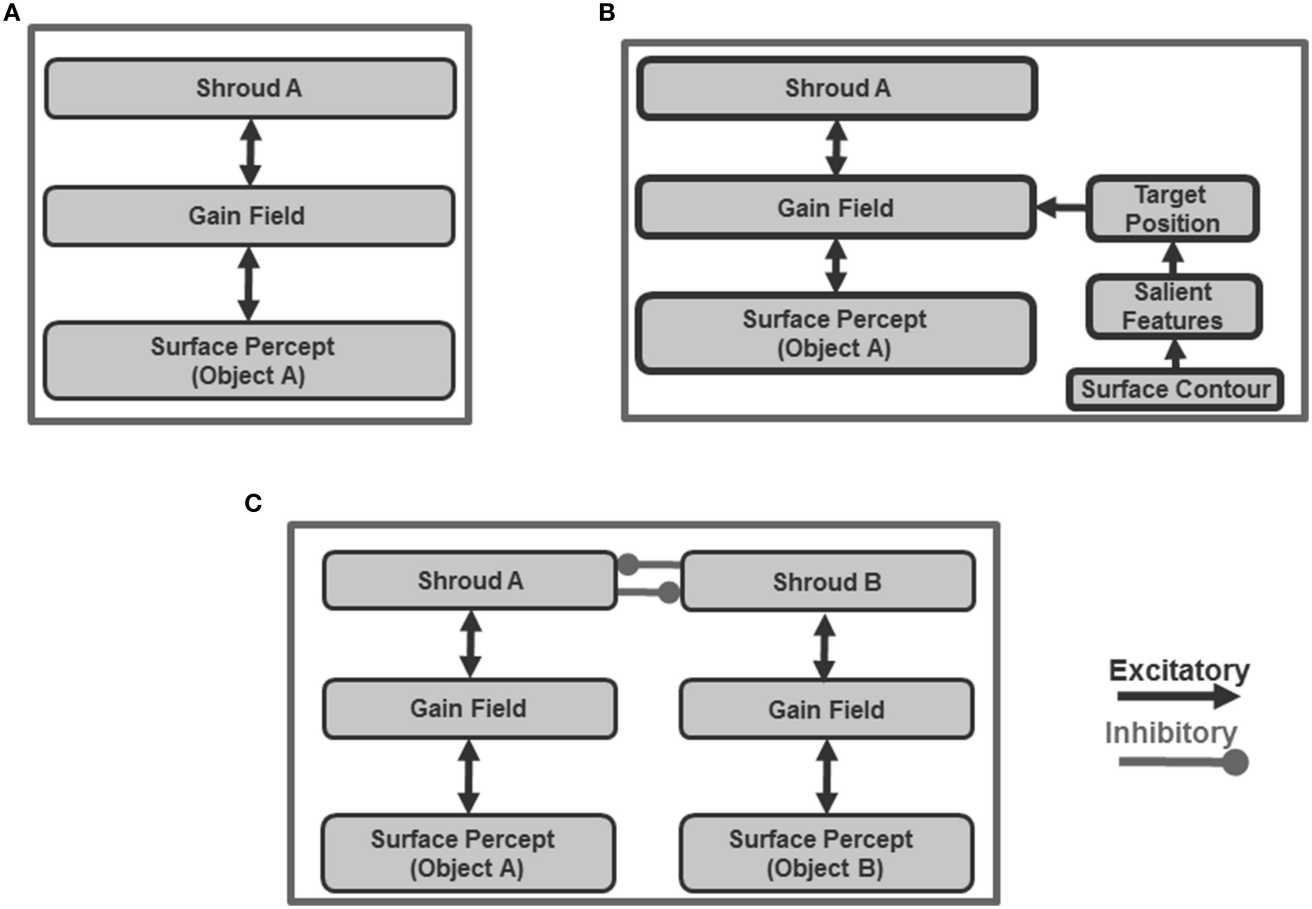
**Schematic for surface-shroud resonance through a feedback interaction between a retinotopic binocular surface and a head-centered spatial attentional shroud. (A)** In the absence of any eye movement to a new target position, the gain fields maintain the stable object shroud of a given object surface. **(B)** When a surface contour is contrast-enhanced to localize salient features (Equation 45), and the position of the most salient feature is chosen as the next target position signal (Equation 67), the gain field is predictively remapped by the target position corollary discharge signal before the corresponding saccadic eye movement occurs (Equation 56), with the result that the shroud retains its stability across eye movements. While the shroud remains active and spatial attention remains focused on a single object surface, the eyes can explore different views of the object, and the What stream of ARTSCAN can learn multiple view-selective object categories and associatively link them to an emerging view-invariant object category. **(C)** If the currently attended shroud collapses, competition across the spatial attention layer (Equation 51)nables another shroud to win the competition and to focus object attention upon the corresponding object surface.

Each eye movement target signal that is derived from a surface contour generates a gain field that maintains a stable shroud in head-centered coordinates as the eyes move (**Figure 5**). These outflow movement commands thus control predictive remapping that maintains attentional stability through time. The stable shroud, in turn, can maintain persistent inhibition of the category reset mechanism as the eyes explore the object and the brain learns multiple view-specific categories of it (Figure 1).

## 3. 3D ARTSCAN model

The 3D ARTSCAN model unifies properties of the ARTSCAN, 3D LAMINART, and aFILM models in a way that is compatible with the pARTSCAN and ARTSCAN Search models. The model does not include the log-polar transformation of cortical magnification, however. This simplification reduces the computational burden in its simulations due to the need to transform binocular inputs into 3D boundary and surface representations that are preserved during eye movements.

### 3.1. Retinal adaptation

Two stages of retinal adaptation (Figure 2; Section 5.1 Equations 1–8) are implemented from the aFILM model of Grossberg and Hong (2006): light adaptation at the outer segment of the photoreceptors and spatial contrast adaptation at the inner segments of photoreceptors. In the outer segment of the photoreceptors, intracellular gating mechanisms such as calcium negative feedback occur (Koutalos and Yau, 1996). This process facilitates light adaptation *in vivo*, by shifting the operating range of the photoreceptor to adapt to the ambient luminance of the visual field. Spatial contrast adaptation at the inner segments of photoreceptors occurs through light adapted inputs from the outer segment, with negative feedback from the horizontal cells (HC) that modulate the influx of calcium ions and control the amount of glutamate release from the photoreceptor terminals (Fahrenfort et al., 1999). The HC network computes spatial contrast using gap junction connections (syncytium) between the HCs. The permeability of the gap junctions between HCs decreases as the difference of the inputs to the coupled photoreceptors increases, and the HCs in the light and dark image regions deliver different suppressive feedback signals to the inner segments of the photoreceptors to properly rescale the inputs that have too much contrast. For simplicity, only gap junction connections between nearest neighbor cells are considered.

During active scanning of natural images with eye movements, the scanned image intensities can vary over several orders of magnitude (Rieke and Rudd, 2009). The model retina uses these two different mechanisms to map widely different input intensities to sensitive, and therefore discriminable, portions of the response range.

### 3.2. LGN polarity-sensitive ON and OFF cells

The LGN ON and OFF cells normalize the adapted contrast and brightness information of the input pattern from the retina using on-center off-surround shunting networks which are solved at equilibrium for computational speed (Figure 2 and Equations 9–12). LGN ON cells respond to image increments (Equation 13) whereas OFF cells respond to image decrements (Equation 14). These single-opponent cells generate output signals that compete at each position, thereby giving rise to double-opponent ON and OFF cells (Equations 15, 16).

### 3.3. Boundary processing

The output signals of the double-opponent ON/OFF LGN cells are the inputs to simple cells that respond selectively to one of four orientations (Equation 17). Simple cell output signals are pooled over all orientations and opposite contrast polarities to create polarity-insensitive complex cell boundaries (Figure 2 and Equation 21). The simplification of pooling over orientation was done because the model is not used to simulate any polarity-specific interactions.

Both monocular and binocular boundaries are needed to generate depthful representations of object boundaries during biological vision (Nakayama and Shimojo, 1990; McKee et al., 1994; Smallman and McKee, 1995; Cao and Grossberg, 2005, 2012). The retinotopic monocular boundaries (Figure 3 and Equation 22) are computed using bottom-up inputs from complex cells (Equation 21). Because they are computed in retinal coordinates, these boundaries are reset whenever the eyes move to fixate a different scenic position. The retinotopic monocular boundaries are also modulated by top-down signals from invariant monocular boundaries (Equation 26) that are not reset by an eye movement. This modulation facilitates predictive remapping. Invariance is achieved using a gain field (Equations 28–32); see Figure 3.

The invariant monocular boundaries (Equation 26) are derived from the retinotopic monocular boundaries (Equation 22), but are computed in head-centered coordinates that are invariant under eye movements. Before the eyes move, the invariant boundaries represent the same positions as the retinotopic boundaries (Equations 24, 25). The invariant monocular boundaries of a stationary object are, however, not reset when the eyes move. They derive their stability due to updated gain field signals that are derived from the next eye movement command even before the eyes actually move to the commanded position. Such predictive remapping of the invariant monocular boundaries to continuously represent the monocular boundaries in head-centered coordinates enables them to be maintained even while the retinotopic boundaries are reset.

The eye movement command is computed from surface contour signals (Sections 3.4–3.6) that are derived from the attended object surface (Figures 1, 4) and that strengthen the boundaries that formed them. Moreover, when the contrast of a surface is increased by feedback from an attentional shroud, the surface contour signals increase, so the strength of the boundaries around the attended surface increase also.

Surface contour signals also activate a parallel, corollary discharge, pathway that projects to the salient features processing stage (Figure 4). In order to compute the position of the next eye movement, these salient features signals are contrast-enhanced by an on-center off-surround network until the most active position is chosen as the next target position. The salient features of an attended surface have an advantage in this competition because they are amplified by shroud-to-surface-to-surface contour feedback.

This target position signal is used both to determine the target position of the next eye movement and to update gain fields that predictively remap retinotopic left and right monocular boundaries into invariant left and right monocular boundaries that remain continuously computed even during eye movements (Figure 3).

The invariant monocular boundaries (Figure 3 and Equation 26) for a given object are fused to yield invariant binocular boundaries (Figure 3 and Equation 33). Because of their computation from invariant monocular boundaries, the invariant binocular boundaries are also maintained as the eyes move. This maintained fusion is a main functional goal of the predictive remapping, since it enables the object percept to persist during eye movements. The fused binocular boundaries, in turn, modulate the activities of the invariant monocular boundaries and thus the activity of cells in the retinotopic boundary layer via top-down feedback through the gain field (Figure 3). This top-down modulatory feedback from the invariant binocular boundary to the invariant monocular boundary ensures that any change or collapse in the invariant binocular boundary activity is propagated back to the retinotopic boundaries (Figure 3).

In the brain, binocular fusion of monocular left and right boundaries tends to occur only between edges with the same contrast polarity (*same-sign hypothesis*; Howard and Rogers, 1995; Howe and Watanabe, 2003) and approximately the same magnitude of contrast (McKee et al., 1994). This constraint naturally arises when the brain fuses edges that derive from the same object in the world, and helps the brain to solve the classical *correspondence problem* (Julesz, 1971; Howard and Rogers, 1995). The model satisfies this constraint through interactions between excitatory and inhibitory cells (Equation 33) that are proposed to occur in layer 3B of cortical area V1 (Grossberg and Howe, 2003; Cao and Grossberg, 2005, 2012). These interactions endow the binocular cells with an *obligate property* (Poggio, 1991) whereby they respond preferentially to left and right eye inputs of approximately equal contrast (Equations 34, 35).

The original ARTSCAN model used gain fields only to predictively update the head-centered representations of attentional shrouds. The current model uses gain fields at several processing stages (Figures 3, 4). They ensure that stable fusion of 3D binocular boundaries and surfaces is maintained in head-centered coordinates as the eyes move. The weights between the gain field neurons and the invariant boundary neurons are presumably learned. For simplicity, only the end product of the learning process, as suggested by Pouget and Snyder (2000), was used in the 3D ARTSCAN model.

### 3.4. Surface processing

**Figure 6:**
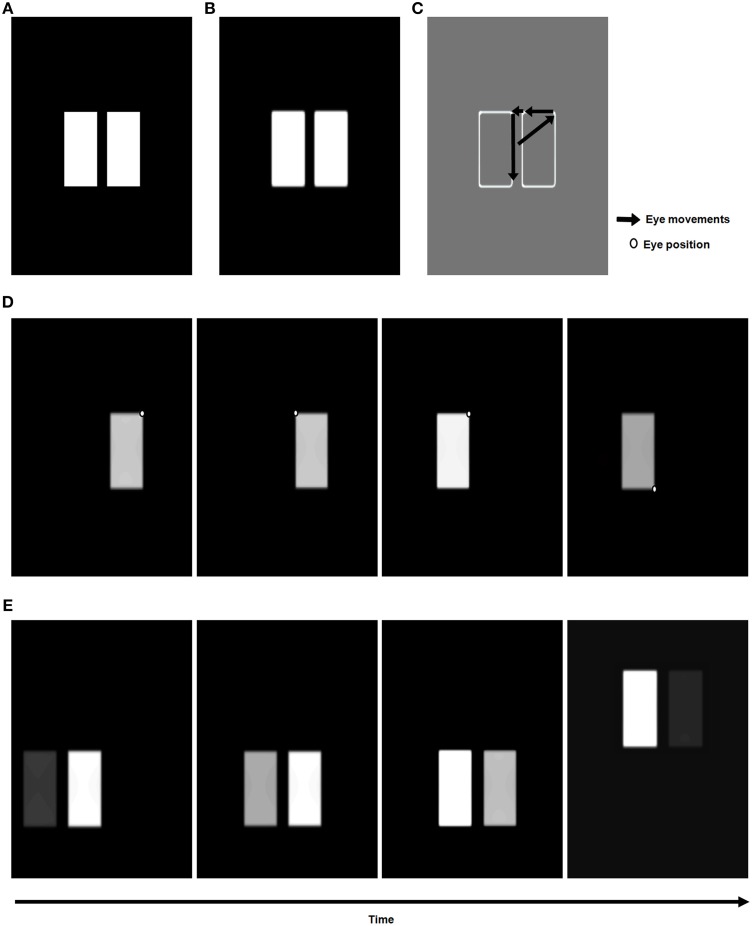
**Model simulations of the 3D ARTSCAN model with simple homogenous surfaces showing stability of binocular surface fusion. (A)** The retinal input (*I*) (Equations 1–3) is a scene containing only two simple objects: two homogenously filled rectangles. This retinal image is presented monocularly to both the eyes. All simulation results are shown for far allelotropic shifts of+3^*o*^. **(B)** In the absence of any eye movements, an initial binocular surface percept (*S*^*b*^) (Equation 41) is formed through the mechanisms of the pre-attentive processing stage for boundaries and surfaces (Figures 2, 3). **(C)** The surface contour map (*C*) (Equation 45) with a cumulative record of all the eye movements to target positions (Equation 66) made within and across the object surfaces is shown. **(D)** As an initial surface percept is formed, competition in the spatial attention map helps to choose a winning attentional shroud (*A*) (Equation 51). The shroud is represented in head-centered coordinates. The eye movements are initiated to salient target positions on the surface contour of a given object surface. In this simple stimulus, the salient features in the surface contours are always one of the corners of the rectangles. The first such surface shroud is activated with an eye movement to the top right corner of the rectangle on the right. Over time, a new target position (dots at rectangle corners) is chosen within or outside the object surface and the next saccade is made. **(E)** The fused binocular surface percept (Equation 41) after each eye movement to a salient feature is shown. Despite eye movements and the collapse of one surface shroud leading to another, the overall binocular surface percept is maintained in retinotopic coordinates. The active surface-shroud resonance enhances the brightness of the attended surface. See Section 4.1 for details.

**Figure 7:**
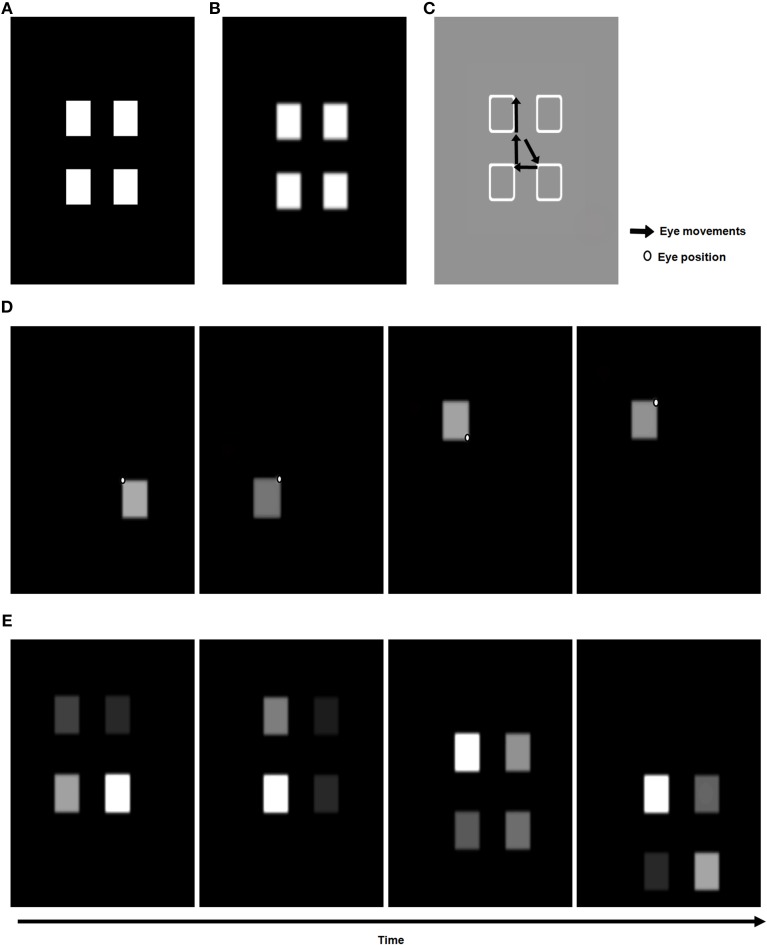
**Model simulations of the 3D ARTSCAN model showing stability of binocular surface fusion with four homogenous objects**. This simulation illustrates that the model scales robustly without any parameter changes. The simulation environment and results are similar to those shown in Figure 6.

**Figure 8:**
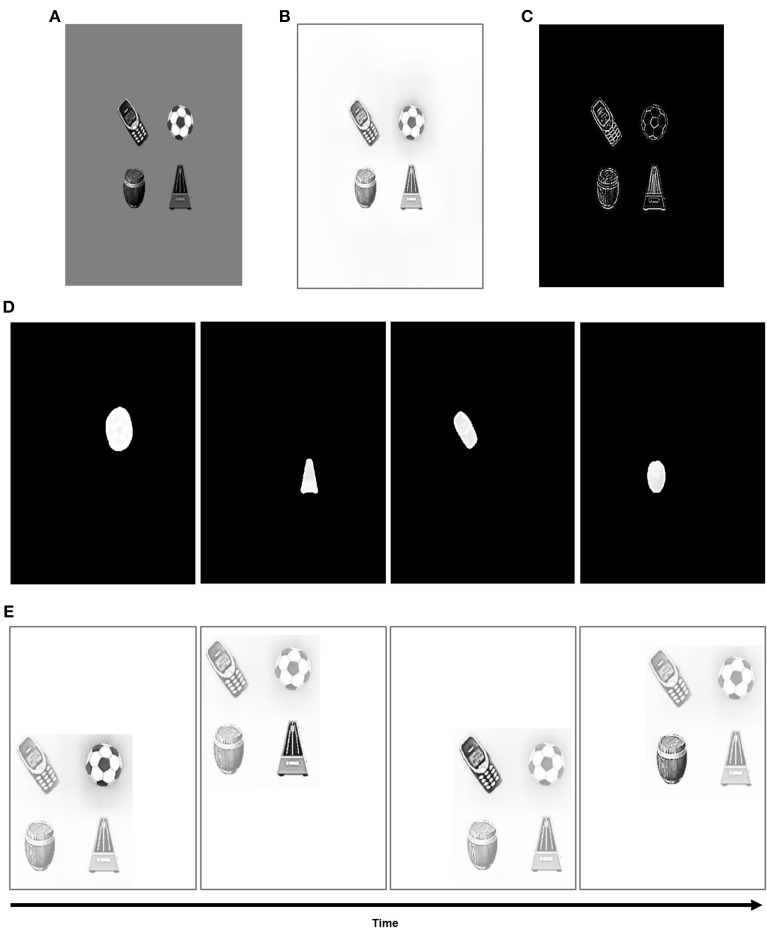
**Model simulations with natural objects showing binocular surface stability**. The results are presented similar to those in Figures 6, 7. The input consists of four non-overlapping grayscale objects with uniform and noiseless gray backgrounds from the Caltech 101 image database (Fei-Fei et al., 2004). The pre-attentive processing stages of the model enabled both the fusion and perceptual quality, including adaptation of ambient illumination, of the binocular surface percepts. Using ON and OFF channels for both boundary and surface representations (e.g., Equations 13–16) improved the perceptual quality of the attended surfaces. **(A)** Input *I* to the system. **(B)** Initial binocular surface percept *S*^*b*^ (Equation 41). **(C)** Surface contour map *C* (Equation 45). **(D)** Attentional shrouds *A* (Equation 51) over time. **(E)** The activity of the binocular surface percept (*S*^*b*^) over time. Several saccades were made within each object's surface contour before moving to the next object. Detailed temporal dynamics of activity of attended shrouds and surfaces are shown in **Figures 10–13**.

**Figure 9:**
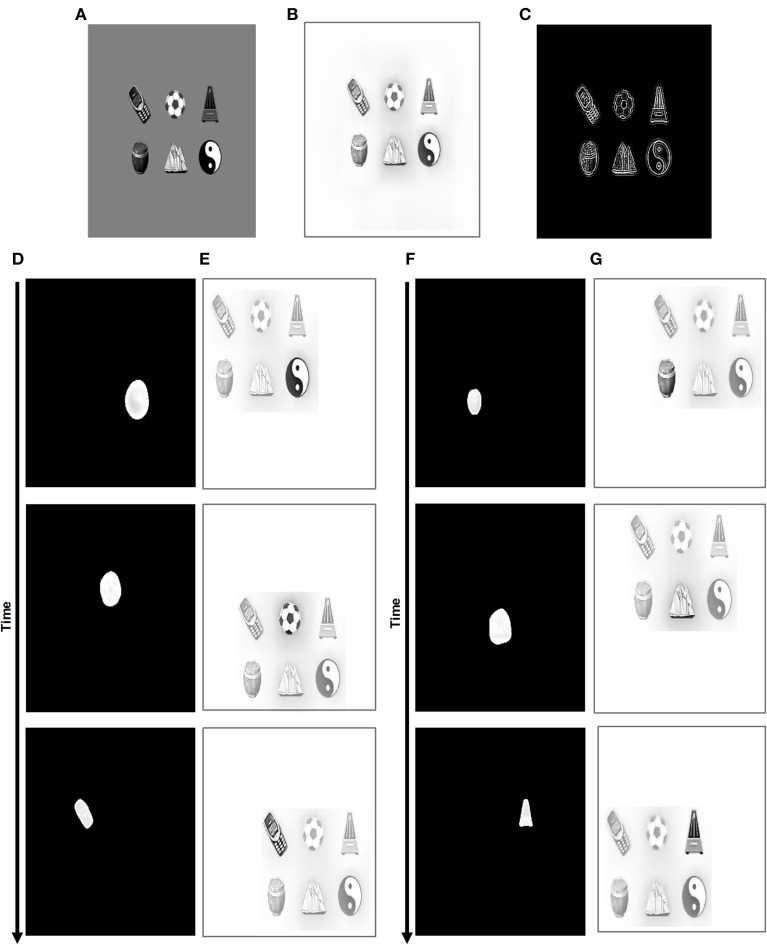
**Model simulations with an increased number of natural objects**. The stimulus and results presented are similar to those in Figure 8, except that the number of objects in the scene is increased to six. **(A)** Input. **(B)** Initial binocular surface percept. **(C)** Surface contour map. (**D,F**) Attentional shrouds over time. (**E,G**) Activity of binocular surface percepts over time.

The invariant binocular boundaries help to maintain the surface representations of stationary objects during eye movements. This is proposed to occur as follows:

Bottom-up inputs from double-opponent ON and OFF cells (Figure 2 and Equations 15, 16) trigger monocular surface filling-in via a diffusion process (Figure 4 and Equation 36), which is gated (Equation 37) by the retinotopic monocular object boundaries (Equation 22) that play the role of filling-in barriers (Grossberg and Todorović, 1988; Grossberg, 1994). The model computes filled-in binocular surfaces in separate double-opponent ON and OFF Filling-In Domains, or FIDOs (Equations 38–40). The final binocular percept is computed as the rectified sum of the ON and OFF FIDO activities [Equation (41) and Figures 6–**9** for simulation results]. This computation enables both light and dark filled-in surfaces to attract spatial attention in a surface-shroud resonance (see Figure 4).

The monocular and binocular FIDOs are computed in retinotopic coordinates, corresponding to the percept that objects that are seen with coarse spatial resolution when the fovea looks elsewhere are seen with cortically-magnified high acuity when they are themselves foveated. The surface contour signals that are derived from these filled-in surfaces are also computed in retinotopic coordinates. These surface contour signals are used to compute the eye movement signals that can command the eyes to move the correct direction and distance to foveate the commanded new fixation position. Aspects of how this happens have been simulated in neural models of saccadic eye movements (e.g., Grossberg et al., 1997; Gancarz and Grossberg, 1998, 1999; Silver et al., 2011).

On the other hand, the invariant binocular boundaries that maintain their fusion across eye movements are computed in head-centered coordinates, even though the monocular left and right boundaries on which they build are initially computed in retinotopic coordinates. Gain fields at several processing stages (Figures 3, 4) cause predictive remapping between these several retinotopic and head-centered representations to maintain binocular fusion of the head-centered boundary representations while eye movements occur.

The head-centered invariant binocular boundaries (Equation 33) regulate surface filling-in within the two retinotopic monocular FIDOs (Figure 4 and Equations 36, 37), which in turn form retinotopic binocularly-fused, or binocular, surface percepts (Figure 4 and Equations 38–40). The head-centered binocular boundaries are converted into retinotopic binocular boundary signals (Equation 40) via gain fields (Figure 4 and Equations 42–44) before they interact with the retinotopic monocular FIDOs. The retinotopic binocular surface percept can support a conscious percept of visible 3D form. Such a consciously seen surface percept in depth is maintained across eye movements due to the predictive remapping of their supporting boundaries by gain fields which occurs at several processing stages (Figure 4 and Equation 38).

The retinotopic binocular surfaces generate surface contour output signals (Figure 4 and Equation 45) through contrast-sensitive shunting on-center off-surround networks (Equations 46, 47). The surface contour signals (Equation 45) provide feedback (Equation 40) to the head-centered binocular boundaries (Equation 33) after being converted back to retinotopic coordinates by gain fields (Figure 4 and Equations 48–50). The surface contour signals from a surface back to its generative boundaries strengthen consistent boundaries, inhibit irrelevant boundaries, and trigger figure-ground separation (Figure 4; Grossberg, 1994; Kelly and Grossberg, 2000). The feedback interaction between boundaries, surfaces, and surface contour signals is predicted to occur between V2 pale stripes and V2 thin stripes.

The coordinated action of all these gain fields acting between boundaries and surfaces, taken together with the surface-based spatial attentional shroud, achieves predictive remapping of the binocularly fused and attended surfaces. See Section 5 for details.

Although the surface filling-in here is modeled by a diffusion process, as in Cohen and Grossberg (1984) and Grossberg and Todorović (1988), Grossberg and Hong (2006) have modeled key properties of filling-in using long-range horizontal connections that operate several orders of magnitude faster than diffusion. Both processes yield similar results at equilibrium.

### 3.5. Spatial shrouds

A surface-shroud resonance fixes spatial attention on an object that is being explored with eye movements. The spatial attention neurons interact via recurrent on-center off-surround interactions (Equations 51–55) whose large off-surround enables selection of a winning attentional shroud. The recurrent on-center interactions enhance the winning shroud, and enable this shroud to remain active as other attentional neurons are persistently inhibited. Top-down attentional feedback from the resonating shroud (Equation 56) increases the contrast of the attended surface (Equation 39).

Such a resonance habituates through time in an activity-dependent way (Equations 51, 61; Grossberg, 1972). Winning shrouds will thus eventually collapse, allowing new surfaces to be attended and causing inhibition of return (IOR). In addition, when a shroud collapses sufficiently during the first moments of a spatial attentional shift, a transient burst of activation by a reset mechanism (Equations 62, 63) helps to complete the collapse of the shroud (Equation 51), as well as to reset the invariant object category in the What stream.

As noted above, object surface input is combined with eye position signals via gain fields to generate a head-centric spatial attentional shroud in the parietal cortex (Figures 4, 5). Such gain field modulation is known to occur in posterior parietal cortex (Andersen and Mountcastle, 1983; Andersen et al., 1985; Gancarz and Grossberg, 1999; Deneve and Pouget, 2003; Pouget et al., 2003). The inputs from the gain fields (Equations 56–60) activate attentional interneurons (Equation 55) that interact through recurrent excitatory signals with attentional cells that excite and inhibit each other via a recurrent on-center off-surround network whose cells obey membrane equation, or shunting, laws (Equation 51).

### 3.6. Eye signals

The eye movement signals serve a major role in predictive remapping of boundaries, surfaces, and shrouds. They also determine the object views that will be attended, and thus which view-specific categories will be learned and associated with the emerging view-invariant object category. The eye movement signals are generated from the surface contour signals (Equation 45) that are derived from the currently active surface-shroud resonance. Surface contour signals tend to be larger at high curvature points and other salient boundary features due to the contrast-enhancing on-center off-surround interactions that generate them from filled-in surface lightnesses and colors. The surface contour signals are further contrast-enhanced to choose the position with the biggest activity, using a recurrent shunting on-center off-surround network (Equations 64–66). This transformation from surface contours to the next eye movement target position is predicted to occur in cortical area V3A (Nakamura and Colby, 2000; Caplovitz and Tse, 2007). These eye movement signals are used to predictively update all the gain field signals (e.g., Equation 48), even before they generate the next saccadic eye movement. The chosen eye movement signal (Equation 66) habituates in an activity-dependent way (Equation 65) and hereby realizes an inhibition-of-return process that prevents perseveration on the same eye movement choice, thereby enabling exploration of multiple views of a given object. See Section 5 for details.

## 4. Simulation results

The entire input visual field is a 3000 × 3000 pixel grid with coordinates (*i, j*) and input intensity *I*_*ij*_. Each pixel step corresponds to a distance of 0.01^*o*^ in visual space, so that each input spans 30^*o*^ × 30^*o*^ in Cartesian space. All object surfaces in the stimulus are within 5^*o*^ on either side of the fixation point. Eye movements were controlled to be within 10^*o*^ of the entire visual field—that is, within the parafoveal region—in order for binocular fusion to be possible. In order to simulate the effects of binocular inputs, the simulations were performed with the monocular inputs shifted with respect to one another by +3^*o*^ (allelotropic far shift). Thus, the inputs to the left and right eye are *I*^*l*^_(*i* + 3^*o*^)*j*_, and *I*^*r*^_(*i* − 3^*o*^)*j*_, respectively. Binocular fusion also works for other allelotropic shifts, far and near, within the range of binocular fusion, as demonstrated in Cao and Grossberg (2005). The range of values of the allelotropic shift *s*, and thus the number of depth planes simultaneously represented in the 3D ARTSCAN model, are {+8^*o*^, +3^*o*^, 0^*o*^, −3^*o*^, −8^*o*^}. The model can readily be extended, without a change of mechanism, to represent any finite number of depth planes. In all the simulations, the initial fixation point was not on any object and was at the center of the visual field. The simulations show how the model's disparity sensitivity to the monocular left and right eye inputs leads to selective activation of the depth plane that is represented by the allelotropic far shift.

### 4.1. Simulations of binocular fusion of homogeneous surfaces

The first simulation tested the ability of 3D ARTSCAN to maintain stable binocular fusion using rectangular-shaped objects as the eyes explored them in a scene. The input consisted of a scene with either two homogenously filled rectangles of equal size (Figure 6A) or four homogeneously filled squares (Figure 7A) on either side of the initial eye fixation point before any eye movements occurred. Each of the rectangles in Figure 6A is 300 × 400 pixels in size. The square stimuli in Figure 7A are each 200 × 200 pixels. The pixellated images are converted into a rectilinear grid in terms of degrees of visual angles as described earlier.

After the initial binocular surfaces are computed, the surface contour map (Equation 45) is also computed, and is shown in Figures 6C, 7C before any eye movements occur. Due to the contrast-sensitive on-center off-surround interactions that generate surface contours from successfully filled-in surfaces, the positions of highest activity (salient features) occur at the corners of the rectangles. When the maximum activities are chosen by a subsequent on-center off-surround network (Equation 66), they determine the targets of the eye movements, which are shown as black arrows. In Figure 6C, the chosen salient feature initiates the first predictive eye movement to the top right corner of the rectangle on the right, consistent with the fact that the rectangle on the right is part of an active surface-shroud resonance (first panel, Figure 6D). Similarly, for the stimulus with four squares, the first eye movement is initiated to the top left corner of the bottom right square (Figure 7C) after the spatial attentional shroud is formed over the corresponding square surface (first panel, Figure 7D). As the eyes continue to move, the scene representation and perceptual stability of the fused binocular surfaces are maintained due to the predictive remapping of the boundaries and surfaces by the gain fields, which ensure that fusion is maintained as the eyes move to the next location. Figures 6D, 7D show the activities of the head-center shrouds, and Figures 6E, 7E show the activities of the corresponding surface representations, of the rectangles and squares through time. When spatial attention is focused on a particular surface as part of a surface-shroud resonance, its activity is enhanced. This is seen in the first panel of Figure 6E, where the rectangle on the right is more active (brighter) than the rectangle on the left. Similarly, the square on the bottom right is more active than others in Figure 7E. This is the fused binocular surface percept and is always in retinotopic coordinates. The attentional shrouds are computed in head-centered coordinates.

As the eyes freely scan the scene, they make several saccades within and across the different object surface contours. As this happens, spatial attention moves from one object, disengaging before engaging another object, based on the salient features in the surface contour map (see Figure 5). A temporal evolution of the spatial attention and binocular percepts are shown from left to right in Figures 6D,E, 7D,E, respectively, for the two stimuli. Before the eyes can move from one object to the other, the currently active attentional shroud begins to collapse due to habituation (Equation 61), which leads to its reset (Equation 62). Multiple saccades move sequentially to the most salient positions on one object's surface contours before moving onto another object's surface contours.

These simulations establish a proof of concept that the extension of the ARTSCAN model to the 3D ARTSCAN model maintains stable fusion of binocular surfaces as the eyes explore them and other objects in their vicinity.

### 4.2. Simulations of binocular fusion of natural objects

Simulations were also carried out using 3D scenes with natural objects in them. For this set of simulations, grayscale images of objects from the Caltech 101 dataset (Fei-Fei et al., 2004) were used. The image backgrounds are a uniform gray and do not have any noise or texture. Each object is 100 × 100 pixels in size. The objects were tiled on the visual field, and two sets of stimuli with four (Figure 8A), and six (Figure 9A) objects were used to test the system's robustness and scalability to more realistic scenes. These pixellated images were rescaled to a rectilinear grid into degrees of visual field, as described earlier. The naturally occurring objects used in the simulations are “cell phone,” “soccer ball,” “metronome,” “barrel,” “yacht,” and “yin yang.”

The pre-processing stages for the natural objects are the same as for the rectangular and square stimuli in Figures 6, 7. The initial binocular surface percept that is represented in retinotopic coordinates is shown in Figures 8B, 9B for the four and six image stimuli, respectively.

The surface contour maps for the natural objects, before any eye movements occur, are shown in Figures 8C, 9C. These simulation figures show the results of when the eyes move from one object's surface contour to the other after the shifting of attentional shrouds. The maintenance of binocular fusion as the eyes move across a single object's surface, followed by shroud collapse and an eye movement to another object, are explained, with simulations, in the remainder of this section and in Section 4.3.

In Figure 8, the first eye movement is made to the soccer ball. Thus, the first spatial attentional shroud is linked to the soccer ball (first panel, Figure 8D). After several saccades explore the soccer ball using its surface contour map to determine salient saccadic target positions, the shroud begins to collapse and spatial attention begins to shift to the metronome as the next eye movement is made to a position chosen from the metronome's surface contour (second panel, Figure 8D). This process then proceeds to the cell phone (third panel, Figure 8D) and then finally to the barrel (fourth panel, Figure 8D). Several saccades are made within each object, thus exploring the object and learning invariant object categories for it (Fazl et al., 2009; Grossberg, 2009; Cao et al., 2011), before moving onto the next object. During all these saccadic eye movements within or across objects and shifts in attention across objects, all the binocular surfaces are maintained in fusion in retinotopic coordinates (Figures 8E, 9E,G). Each panel that illustrates the binocular percept shows enhanced activity of the currently attended object surface.

**Figure 10:**
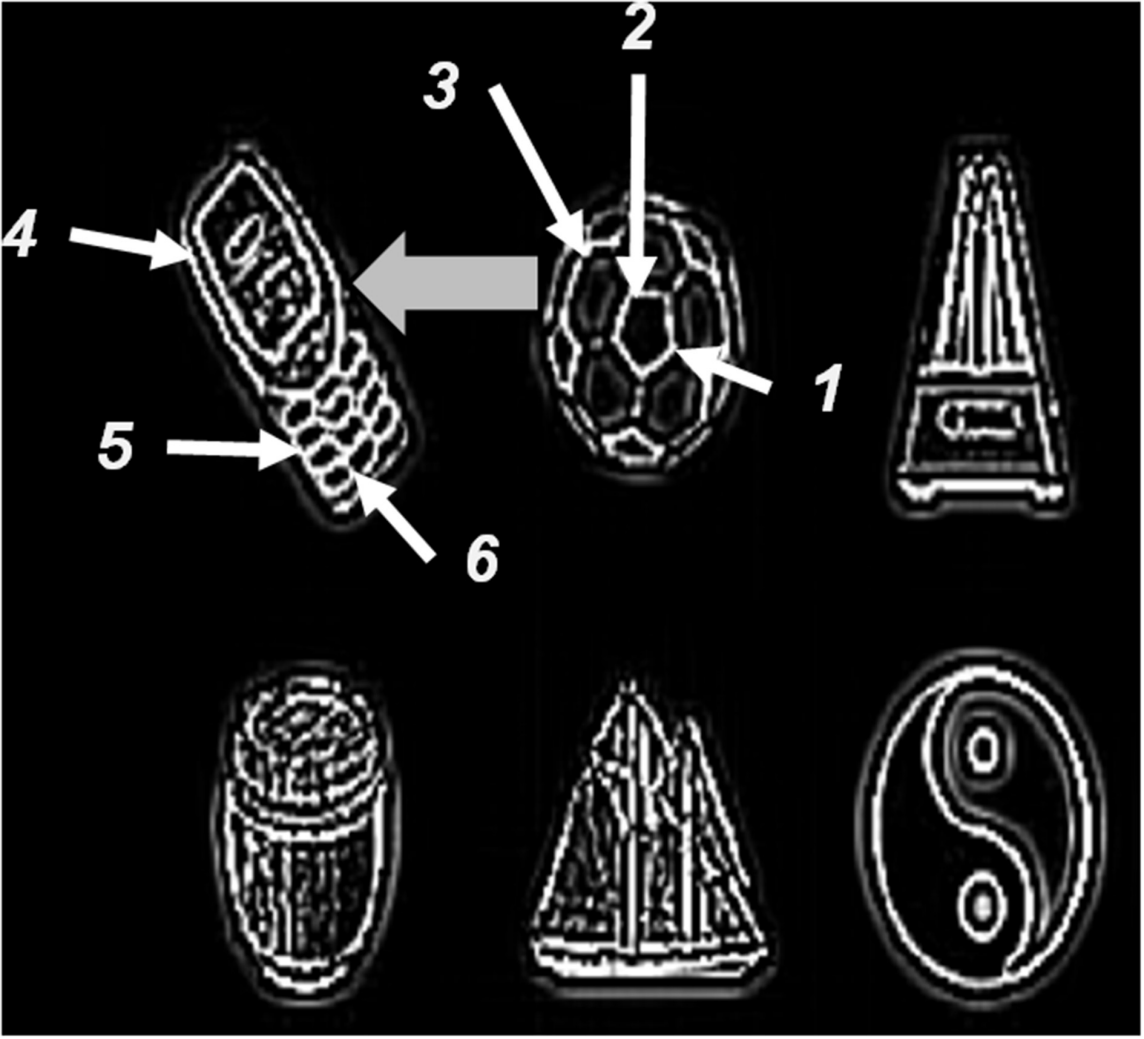
**Surface contour activity *C* (Equation 45) with attention first maintained on the soccer ball, followed by a then shift in attention to the cell phone**. Saccades to target positions marked “*1*,” “*2*,” and “*3*” are made within the soccer ball. Saccades to target positions marked “*4*,” “*5*,” and “*6*” are made within the cell phone after a shift in attention. The thick gray arrow marks the shift in attention from the soccer ball to the cell phone following parietal reset (see Section 4.3 for details).

The same experiment was repeated with more stimuli (six instead of four) in the scene to test the scalability and robustness of the system; see Figure 9. Here, the first predictive eye movement is made to the yin yang symbol (first panel, Figure 9D) as its attentional shroud suppresses the shrouds of the other objects. After a few saccades on the yin yang surface contour, an eye movement is made to the soccer ball surface contour as spatial attention is disengaged from the yin yang and engaged with the soccer ball (second panel, Figure 9D). After this, an eye movement is made to the cell phone surface contour: spatial attention is disengaged from the soccer ball, and engaged with the cell phone (third panel, Figure 9D). This is then followed by an eye movement to the barrel, yacht, and finally to the metronome (panels in Figure 9F). Within each object, several saccades were made before moving onto the next object (see Figure 10).

The binocular surface percept remains fused in retinotopic coordinates while all this change occurs in spatial attention and eye movements. Here again, the perceptual contrast of the attended surface, which is in surface-shroud resonance, is enhanced (Figures 8E, 9E,G). This simulation shows that system properties, using the same set of parameters, are robust in response to variable numbers of natural images. The invariant binocular boundaries were as well maintained in fusion by the predictive remapping signals. These dynamics are elaborated in Sections 4.3 and 4.4.

### 4.3. Simulations of within object eye movements and attention shifts between objects

Sections 4.1–4.2 and Figures 6–9 summarized simulations that illustrate how homogeneous surfaces (rectangles and squares) and natural objects induce surface representations that remain binocularly fused as attention shifts from one object to another during scanning eye movements. Figure 10 describes the surface contours (Equation 45) before any eye movements occurred, as well as six of the eye movement target positions that were determined by the surface contours and which led to eye movements.

When attention is disengaged from the yin yang and shifts to the soccer ball, the fixated eye position (Equation 66) within the soccer ball is marked as “*1*” on the surface contour in Figure 10. The activities of the attentional shroud and the fused binocular surface after the eye position “*1*” is attained are shown in Figures 9D,E (second row), respectively. Following this, two more saccades numbered “*2*” and “*3*” are made to surface contour salient features of the soccer ball (Figure 10). While these saccadic explorations are made within the soccer ball, its shroud starts to collapse due to a combination of inhibition of return and habituation. This disinhibits and triggers the burst of the parietal reset signal (Equation 62), which was thus far inhibited by the active shroud of the soccer ball. This burst of the reset signal collapses the habituating attentional shroud on the soccer ball completely, thus initiating a shift in spatial attention (thick gray arrow) from the soccer ball to the cell phone. Once the spatial shift in attention to the cell phone occurs, the new eye position (Equation 66) within the cell phone is marked as “*4*” on the surface contour (Figure 10). Two saccades numbered “*5*,” “*6*” are next made within the cell phone. The binocular surface percept and attentional shroud activity of the cell phone, for the position marked as “*6*” was shown previously (third panel, Figures 9D,E).

**Figure 11:**
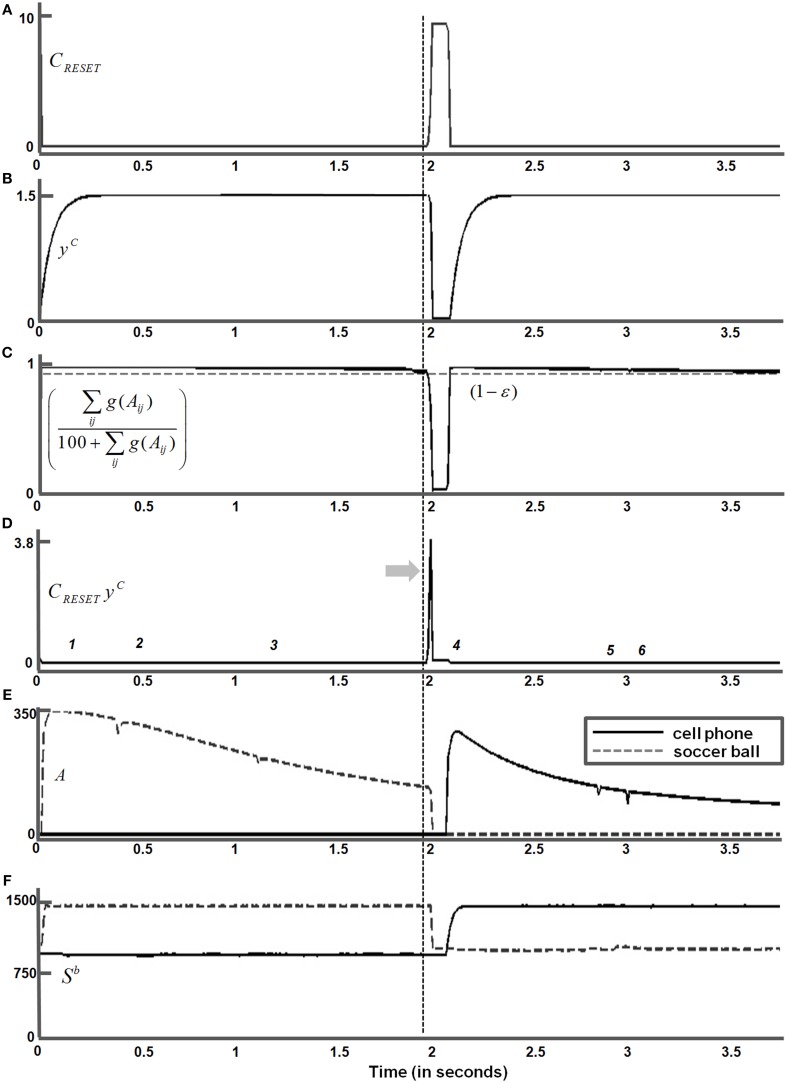
**Temporal dynamics after attention is engaged by the soccer ball and saccades are made within it, followed by a shift in attention to the cell phone and saccades within the cell phone. (A)** Temporal evolution of the parietal reset signal *C*_*RESET*_ (Figure 4 and Equation 62) for the paradigm described in Figure 10. When saccades are made within the attended object, *C*_*RESET*_ remains inhibited, thereby allowing for explorations of different views within the attended object that can be learned and associated with a view-invariant category of the object. A few moments after $\frac{\sum_{ij}g(A_{ij})}{100+\sum_{ij}g(A_{ij})}$ in Figure C crosses beneath the threshold (1 − ε), the parietal reset signal is disinhibited and inhibits the currently active shroud, thereby enabling a shift in spatial attention. The time when *C*_*RESET*_ turns on is marked by the dashed vertical line. When the next winning shroud starts to become active **(E)**, it inhibits the reset signal. **(B)** The habituative neurotransmitter *y*^*C*^ (Equation 63) is at its maximum activity when the reset signal is inhibited. When the reset signal is activated, the transmitter habituates in an activity-dependent way. The net reset signal *C*_*RESET*_ *y*^*C*^ that inhibits the spatial attention map (Equation 51) is therefore transient. An attention shift to a new surface-shroud resonance can hereby develop after it shuts off. When the reset signal is inhibited by the newly active shroud, the habituative neurotransmitter gradually replenishes over time before the next reset event occurs. **(C)** The temporal evolution of the ratio of the attention function $\frac{\sum_{ij}g(A_{ij})}{100+\sum_{ij}g(A_{ij})}$ that is subtracted from the constant threshold (1 − ε) = 0.93 to define the parietal reset signal. As long as the ratio of the attention function remains above the threshold, the reset signal remains inhibited. After the ratio crosses the threshold (marked by the dashed vertical line), the parietal reset signal is turned on. **(D)** The transient reset burst *C*_*RESET*_ *y*^*C*^ inhibits the spatial attention map. **(E)** Temporal evolution of the attentional shrouds *A* (Equation 51) of the soccer ball and cell phone. The reset mechanism does not collapse the shroud when saccades (e.g., “*2-3*” or “*5-6*” in Figures 10, 11D) are made within the surface of an active shroud. The small dips in activity of the active shroud correspond to saccades within the attended object. **(F)** Temporal evolution of the binocular surface percepts *S*^*b*^ (Equation 41). The attended binocular surface activity (dashed curve, soccer ball; solid curve, cell phone) is enhanced by surface-shroud resonance. See Section 4.3 for details.

The temporal evolution of the parietal reset signal (Figure 4 and Equation 62) during these six eye movements (Figure 10) is shown in Figure 11A. A reset signal occurs only when the soccer ball shroud collapses, thereby enabling a spatial attention shift to the cell phone. The eye movements within these objects do not cause a reset signal. The temporal profile of the habituative transmitter (Figure 4 and Equation 63) that gates the parietal reset signal is shown in Figure 11B. The temporal evolution of the ratio $\frac{\sum_{ij}g(A_{ij})}{100+\sum_{ij}g(A_{ij})}$ that is subtracted from the constant threshold (1 − ε) to define the parietal reset signal *C*_*RESET*_ in Equation (62) is shown in Figure 11C. When $\frac{\sum_{ij}g(A_{ij})}{100+\sum_{ij}g(A_{ij})}$ becomes smaller than (1 − ε), *C*_*RESET*_ turns on at the time marked by the dashed vertical line, as in Figure 11A, and the habituative gate begins to decay in an activity-dependent way, as in Figure 11B. As a result, the net reset signal *C*_*RESET*_ *y*^*C*^ in Figure 11D is a transient burst. This transient burst completely inhibits the active soccer ball shroud (dashed line) in Figure 11E via Equation (51). There is a time lag between the activation of successive shrouds, following the collapse of soccer ball shroud and the formation of the cell phone shroud (solid line), that corresponds to the time needed to shift spatial attention between the two objects (Figure 11E). The inhibition of the soccer ball shroud enables the cell phone shroud to win the competition for spatial attention. The binocular surface representation of the cell phone (Figure 11F and Equations 38–41) is then enhanced by top-down excitatory feedback from its shroud as a surface-shroud resonance develops. The newly activated shroud inhibits the tonically active reset signal (Figure 11A) and the habituative transmitter gradually recovers through time (Figure 11B). These dynamics repeat when next reset event occurs.

**Figure 12:**
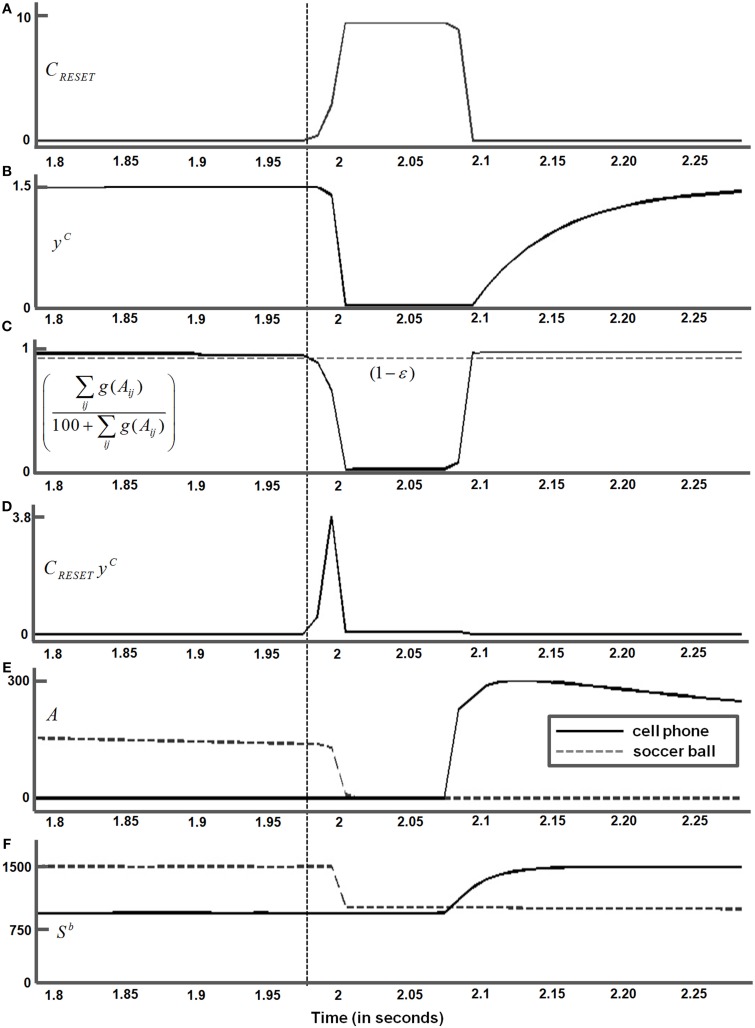
**Temporal dynamics of the plots in Figure 11, but at a finer temporal resolution before, during, and after the transient reset burst**.

Figure 12 presents the evolution of the activities shown in Figure 11 at finer temporal resolution at times just before, during, and after the occurrence of the reset event so that the reader can better appreciate these temporal details. When saccades (e.g., “*2–3*” or “*5–6*” in Figure 10) are made within the surface of an active shroud, they do not cause the reset mechanism to collapse the shroud. The small dips of activity in the active shrouds in Figure 11E correspond to such eye movements within an object. As a result of these saccadic explorations within an attended object, different view-specific categories of the object can be learned and associated with a view-invariant category of the object (see What stream of ARTSCAN in Figure 1).

**Figure 13:**
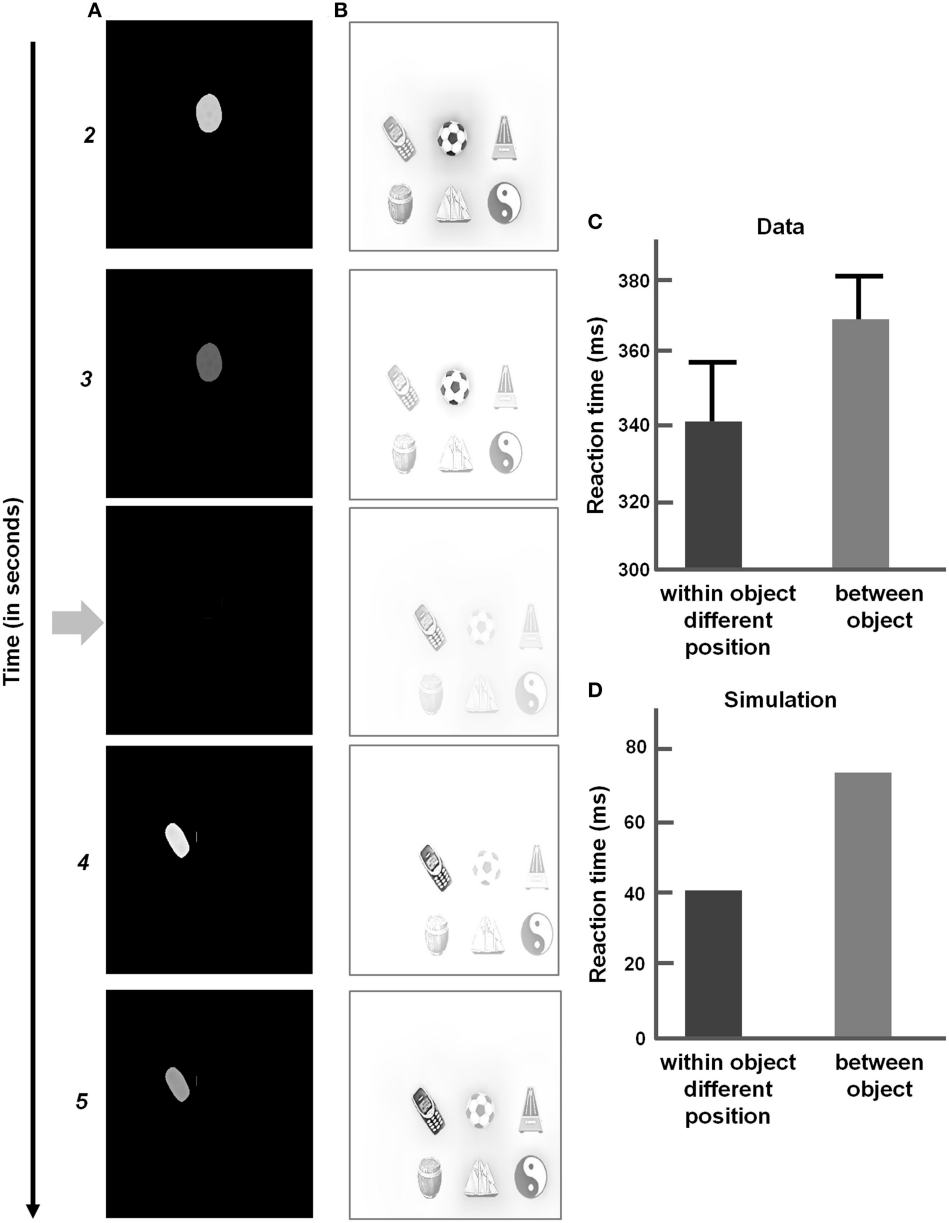
**Snapshots of the attentional shroud and the binocular surface percept during saccades within the soccer ball, followed by a shift in attention to the cell phone and a saccade within it. (A)** Activities of attentional shrouds *A* (Equation 51) in head-centered coordinates after saccades to target positions “*2*,” and “*3*” within the soccer ball, followed by an attentional shift to the cell phone (thick gray arrow), when no shroud is active, after which a cell phone shroud forms around target position “*4*,” and then a saccade occurs within the cell phone to target position “*5*.” **(B)** Corresponding activation patterns of the binocular surface percept (*S*^*b*^) (Equation 41) in retinotopic coordinates. The eye positions and the attentional shift correspond to the paradigm explained in Figure 10 and for the temporal profiles shown in Figure 11 (see Section 4.3 for details). **(C)** Reaction time (RT) data from Brown and Denny (2007) for *within-object different position* (341 ± 9 ms, dark gray), and *between objects* (369 ± 10 ms, light gray) trials. **(D)** Simulations of RTs to object-based attention computed over the spatial attention map *A*. Average RTs to *within-object different position* (40ms, dark gray), and *between objects* (75 ms, light gray) are shown for the complete simulation run in Figure 9. RTs to attend to *within-object* different *positions* are faster than *between objects*, consistent with the data in **(C)** See Section 4.3 for an explanation of why the RT difference matches the data, but the total simulated RTs are 300 ms shorter.

Figure 13 shows the simulated activity profiles of the attentional shroud and binocular surface representations when saccades are made, as summarized in Figure 10, within an attended surface, and after shifts in attention to other surfaces. Figure 13A shows the profiles of the attentional shrouds which are represented in head-centered coordinates, and Figure 13B shows the profiles of the corresponding binocular surface percepts in retinotopic coordinates. The markings “*2*,” “*3*,” “*4*,” “*5*,” and the thick gray arrow on the sides of each pair of panels correspond to the eye positions after each saccade, and the shifts in attention described in Figures 10–12.

Figure 13C shows the average reaction time (RT) data in human subjects of Brown and Denny (2007). Figure 13D shows the average RTs to attend for the simulations shown in Figure 9. Average RTs in the simulations are computed on the spatial attention map(*A*) (Equation 51). The average reaction times for attending *within-object different position* (dark gray bar) after saccades are faster than the average response times for *between-object* (light gray bar) shifts of attention. The average reaction times for *within-object different position* after saccades were calculated as the time it takes the active shrouds to recover from the small dips in activity, corresponding to eye movement made within the object to a different target position (e.g., Figure 11E). The average reaction times for *between-object* shifts in attention were calculated as the time between the complete collapse of the previous shroud and the activation of the next shroud to half its maximum value (Figures 11E, 12E). The investigations of Brown and Denny (2007) showed that between-object shifts of attention take longer than within-object shifts. This within-object advantage occurs because attention need not be disengaged from the object when eye movements to target positions are made inside it. Brown and Denny (2007) also found that shifting attention from an object to another object, or to another position with no object present, takes nearly the same amount of time (369 ± 10 vs. 376 ± 9 ms), concluding that the engagement of attention is not the time limiting step in object-based experiments.

In the ARTSCAN model (cf. Fazl et al., 2009, Figure 1), the RTs for the corresponding simulations were scaled to be equal to the valid trials in the data. The dARTSCAN (cf. Foley et al., 2012) model has generalized ARTSCAN beyond its parietal spatial attentional capabilities to include prefrontal working memory storage, and has thereby extended the Fazl et al. (2009) simulations to quantitatively simulate all of the experimental cases described by Brown and Denny (2007). The 3D ARTSCAN model replicates two of the trial conditions from the Brown and Denny (2007) experiment. The *within-object different position* (341 ± 9 ms, dark gray) and *between-object* (369 ± 10 ms, light gray) RTs in Figure 13C correspond to the invalid within, and invalid between, object trials of the experiment. The simulation RTs of *within-object different position* (40 ms, dark gray) and *between-object* (75 ms, light gray) presented in Figure 13D consistent with the data in Figure 13C. In ARTSCAN and dARTSCAN, trials were run explicitly instructing the system of the prime and cue, followed by a long inter-stimulus interval (ISI) before the target appears and a response is made with the appearance of the target. However, in 3D ARTSCAN, the cue and target selections are internally evaluated from the salient features on the surface contour map without any experimenter supervision, and only the response time is calculated from when the salient feature appears followed by an eye movement to the target position. The RTs shown here are thus 300 ms less than what was reported in Brown and Denny (2007).

### 4.4. Simulations of predictive remapping of binocular boundaries

**Figure 14:**
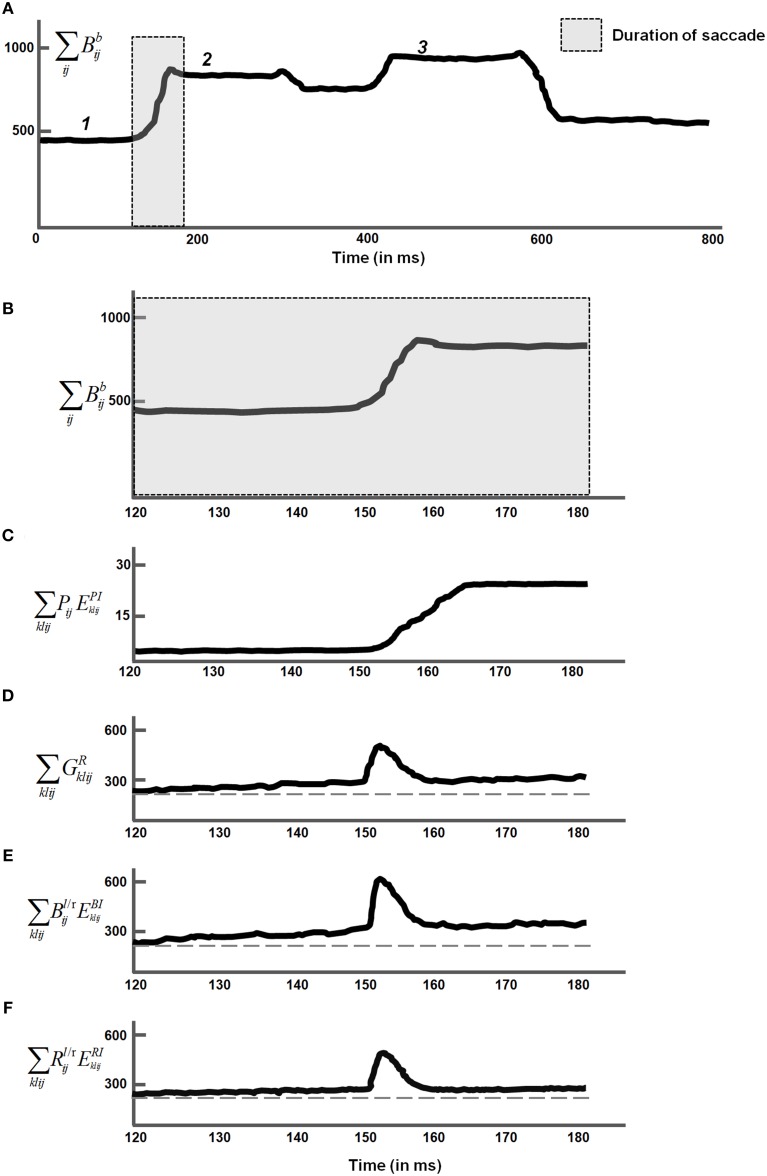
**Predictive remapping of fused invariant binocular boundaries**. The input stimulus is the same as in Figure 9 and the paradigm is from Figure 10. The maintained fusion of boundaries is demonstrated when saccades are made to target positions within one object, in this case, the soccer ball. For convenience, only ON channel (+) responses are shown. The OFF channel (−) responses look similar and thus the +/− superscripts are dropped for convenience. **(A)** Temporal evolution of the fused invariant binocular boundaries ∑_*ij*_*B*^*b*^_*ij*_ (Equation 33) when saccades are made within the soccer ball. The markings “*1*,” “*2*,” and “*3*” correspond to the target positions on the surface contour map shown in Figure 10. The dashed gray box is the duration of the saccade (60 ms) for which the dynamics are presented in (**B–F**). **(B)** Temporal evolution of the invariant binocular boundaries ∑_*ij*_*B*^*b*^_*ij*_ before, during, and after an eye movement to target position “*2*” in Figure 10 following fusion of the invariant monocular boundaries. The dotted gray box shown covers the duration of the saccade shown in **(A)**. Even before the eye movement is completed, there is predictive remapping of the fused boundaries by the boundary gain fields. **(C–F)** show the boundary gain field activity for the left eye (*l*). The right eye profiles are the same. To achieve predictive remapping of the invariant left monocular boundary, the invariant left monocular boundary gain fields *G*^*L*^*l*^^_*klij*_ (Equation 28) are activated by top-down inputs ∑_*ij*_*B*^*l*^_*ij*_ *E*^*BI*^_*klij*_ from invariant left monocular boundaries (Equation 26), eye position signals ∑_*klij*_*P*_*ij*_ *E*^*PI*^_*klij*_ (Equation 66), and bottom-up inputs ∑_*klij*_*R*^*l*^_*ij*_ *E*^*RI*^_*klij*_ from retinotopic left monocular boundaries (Equation 22). **(C)** Temporal profile of the eye position input ∑_*klij*_*P*_*ij*_ *E*^*PI*^_*klij*_. (**(D)** Temporal evolution of the summed invariant left monocular boundary gain field activity ∑_*klij*_*G*^*R*^*l*^^_*klij*_. **(E)** Temporal profile of the invariant left monocular boundary input ∑_*klij*_*B*^*l*^_*ij*_ *E*^*BI*^_*klij*_. **(F)** Temporal evolution of the retinotopic left monocular boundary input ∑_*klij*_*R*^*l*^_*ij*_ *E*^*RI*^_*klij*_. The gray dotted lines in **(D–F)** show the change in activity from baseline. See Section 4.4 for details.

**Figure 15:**
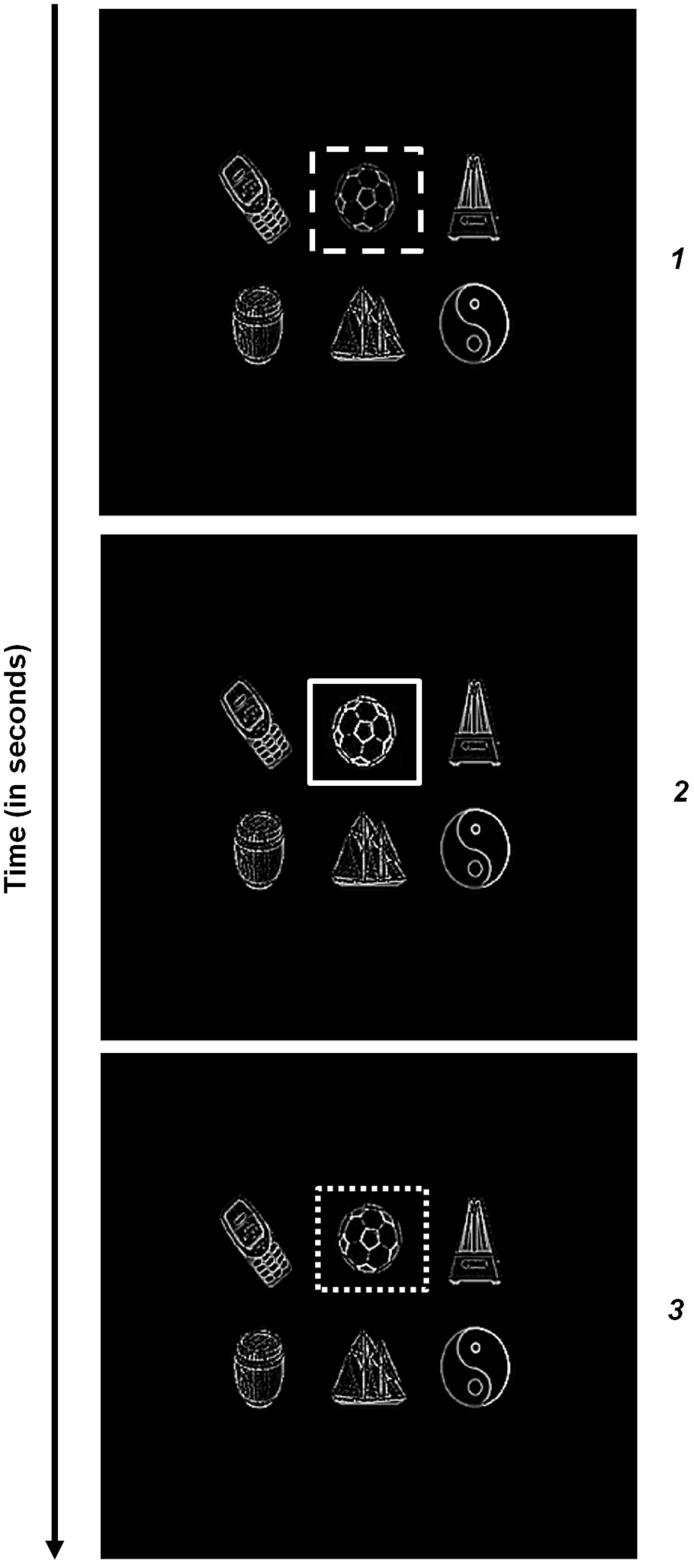
**Snapshot of fused invariant binocular boundaries *B*^*b*^ (Equation 33) of all the objects after saccades to target positions within the attended soccer ball**. Only ON channel invariant binocular boundaries are shown. Following the paradigm in Figure 10, and the temporal profile in Figure 14A, the corresponding fused binocular boundaries are shown after the shift in attention to target position “*1*” followed by saccades to target positions “*2*,” and “*3*” within the soccer ball. All the binocular boundaries are maintained in head-centered coordinates. The activities of the fused soccer ball boundaries are enhanced (“*1*,” dashed box; “*2*,” solid box; and “*3*,” dotted box) as saccades are made to the corresponding target positions. Binocular boundaries of unattended objects remain fused as well. See Section 4.4 for details.

Figures 14, 15 summarize simulations of predictive remapping by gain field modulation to maintain fusion of invariant binocular boundaries during eye movements. The inputs used in this analysis are the same as in previous sections (Sections 4.2–4.3 and Figures 9, 10). The surface contour map from which eye position signals are generated is shown in Figure 10. The temporal dynamics of the predictive remapping of fused invariant binocular boundaries of all the objects are presented in Figure 14 at the position marked “*2*” in Figure 10 while saccadic eye movements are made to the target positions within the soccer ball to positions marked “*1*,” “*2*,” and “*3*.”

Figure 14A shows the temporal profile of the summed response of the fused invariant binocular boundaries (∑_*ij*_*B*^*b*^_*ij*_) (Figures 3, 4, and Equation 33) for all the objects following a shift in attention from the yin yang to position “*1*” within the soccer ball. This is followed by two saccades to target positions “*2*” and “*3*” within the soccer ball. The duration of the saccade from position “*1*” to “*2*” is indicated by the gray dotted box, and is 60 ms. In all plots in Figure 14, only the ON channel profiles are shown. The OFF channel responses look similar. The +/− superscripts are thus dropped for convenience. The summation of the invariant binocular boundary values (∑_*ij*_*B*^*b*^_*ij*_) is plotted to show how the boundaries of all the objects are maintained in fusion while saccades are made to target positions within the soccer ball. This happens because the binocular boundaries are maintained in fusion in head-centered coordinates before the eye movement to the next target position, following predictive remapping of monocular boundaries in head-centered coordinates by monocular boundary gain fields (Equations 28–32). The monocular boundary gain fields are updated by predictive eye signals (Equations 64–66) that are derived from the surface contour map (Equation 45), as illustrated in the remainder of Figure 14. Additionally, the binocular boundaries of the attended object (the soccer ball) are strengthened by top-down feedback from the surface contour map (Equation 45) via gain fields (Equation 48). Thus, in Figure 14A it can be observed that there is an increase in summed activity of all the binocular boundaries by predictive buildup of the boundary gain fields acting on the monocular gain fields (their dynamics are explained in Figures 14C–F). Enhanced activity after the initial buildup for the invariant binocular boundaries of the attended surface (soccer ball) is maintained by its surface contour feedback (see Figure 15 for illustration).

Figures 14B–F show a blown-up time scale (note the finer time scale) of these boundary dynamics achieved by a combination of the gain field activities and how they correlate with gain field predictive dynamics during the duration of the saccade. Figure 14B shows the temporal profile of the invariant binocular boundaries before, during, and after the eye movement from target position “*1*” to “*2*.” This corresponds to the activity of the binocular boundaries shown in the gray dotted box in Figure 14A. Note the buildup and maintenance of the fused binocular boundary activity even before the eye movement (Equation 66) to the target position is completed, which only ends after 180 ms.

The invariant binocular boundaries *B*^*b*^ (Equation 33) are fused from invariant monocular boundaries *B*^*l/r*^_*ij*_ (Equation 26) that are derived from the retinotopic monocular boundaries *R*^*l/r*^_*ij*_ (Equation 22). This transformation from retinotopic to invariant monocular boundaries is achieved through predictive remapping by boundary gain fields (Equations 28–32), which are subsequently fused to yield the binocular boundaries (Equation 33). In Figures 14C–F, only the left monocular ON channel predictive remapping activities are presented. The summed activation patterns for the right monocular ON/OFF channels are exactly the same as that of the left images. In Figures 14D–F, the horizontal gray dashed lines are drawn to show how predictive remapping enhances the activities from before the eye movement to the target position.

Figure 14C plots the summed temporal activity of the eye position signal's *P* (Equation 66) gain modulation, defined as ∑_*klij*_*P*_*ij*_ *E*^*PI*^_*klij*_ [in Equation (28)]. This modulates the boundary gain field in order to achieve predictive remapping of the invariant monocular boundary (see Figure 3). Only one target position is active at any given time and it can be observed that during the period of eye movement, there is a gradual buildup of this activity. Before the eye movement to a target position derived from the salient features is completed, the modulation from the predictive target position signal ensures that the invariant monocular boundaries are remapped to maintain the fusion of the binocular boundaries. The activity of this component is maintained at that level until the next eye movement occurs (here from target position “*2*” to “*3*”).

The temporal evolution of the summed boundary gain field activity *G*^*R*^*l*^^ (Equation 28) as ∑_*klij*_*G*^*R*^*l*^^_*klij*_, responsible for predictive remapping of the invariant monocular boundaries, is presented in Figure 14D. These boundary gain fields are modulated by the bottom-up inputs from retinotopic monocular boundaries (Equation 22), the target eye position signal (Equation 66), and feedback from the invariant monocular boundaries (Equation 26). These gain fields in turn modulate and predictively remap the invariant monocular boundaries (Equation 26) as well as the retinotopic monocular boundaries [Equation (22), also see Figure 3]. In Figure 14D, it can be observed that during the eye movement, there is a predictive buildup of the gain field activity. At the end of the eye movement, the overall gain field activity is enhanced from the initial value as marked by the dashed gray line. The transient increase in activity followed by plateauing is caused by a combination of top-down feedback from the invariant monocular boundaries and the bottom-up retinotopic monocular boundaries.

Figure 14E plots the summed temporal activity of the invariant left monocular boundaries' *B*^*l*^ (Equation 26) gain modulation expressed as ∑_*klij*_*B*^*l*^_*ij*_ *E*^*BI*^_*klij*_ (in Equation 28). Again there is a predictive buildup of this component and, after the transient activation, the activity plateaus. This transient activation is a combination of feedforward retinotopic inputs via the gain fields, followed by modulatory feedback from the fused invariant binocular boundaries to the invariant monocular boundaries. The gray horizontal line clearly shows an enhanced activation of the invariant monocular activation from its initial value before the saccade.

Figure 14F plots the summed temporal activity of the retinotopic left monocular boundaries' *R*^*l*^ (Equation 22) gain modulation ∑_*klij*_*R*^*l*^_*ij*_ *E*^*RI*^_*klij*_ [in Equation (28)]. During the eye movement to the target position “*2*,” there is a buildup of this activity, followed by a transient activity before plateauing. The transient activity is caused by feedback from the invariant left monocular boundary via the boundary gain fields. The invariant left monocular boundaries in turn are modulated by invariant binocular boundaries (Figure 3 and Equation 26). Thus, even before an eye movement is completed to the target position, the boundary gain fields predictively remap the invariant monocular boundaries. These invariant monocular boundaries are fused to yield invariant binocular boundaries, in which the binocular boundaries of the attended object are further strengthened by top-down feedback from their surface contour signals.

Figure 15 shows snapshots of activation profiles of the invariant fused binocular boundaries after a saccade occurs to those target positions (“*1*,” dashed; “*2*,” plain; and “*3*,” dotted box) as shown in Figure 10. Again for convenience, only the ON channel invariant binocular boundaries are shown. It can be observed from the three snapshots in Figure 15 that the binocular boundaries of all the six objects in the scene remain fused after every subsequent eye movement to the three different target positions within the soccer ball. They are also maintained in head-centered coordinates throughout the time when eye movements are made to target positions within the soccer ball. Further, the activity of binocular boundaries of the attended soccer ball surface is enhanced with every eye movement due to surface contour feedback.

## 5. Mathematical equations and parameters

Unless specified otherwise, the equations are all solved dynamically. Symbol *I* is the input image and *I*_*ij*_ is the value of the input image in the visual field at position (*i, j*). The dynamic range of inputs *I*_*ij*_ is [0, 1]. The superscripts *l/r* are used to denote the boundary/surface processing in the left or right eyes, respectively. The superscripts +/− are used to denote ON and OFF processing, respectively. The equations and parameters used for monocular cells that are responsive to the left or right eyes, and for ON and OFF cells are the same in the simulations, unless specified otherwise. The binocular cells/networks have a *b* superscript. The simulations are shown for a single depth with allelotropic shifts of *s* = +3^*o*^ where the neurons are tuned for far disparity. The image input *I*_*ij*_ at position (*i, j*) gives rise to monocular inputs to the left and right eyes equal to *I*^*l*^_(*i* + *s*)*j*_, and *I*^*r*^_(*i* − *s*)*j*_, respectively, for all *i* and *j* that project to the retina. The simulations were carried out in MathWorks (R) MATLAB R2012a (TM) on a Linux GNOME x64 bit machine with Intel Quad-Core (TM)/3.10 GHz/7.7 GB of RAM.

### 5.1. Retinal adaptation

The retinal equations have been adapted from the aFILM model of Grossberg and Hong (2006). The potential ϕ^*l/r*^_*ij*_ at position (*i, j*) of the outer segment of the retinal photoreceptor is simulated by the equation:

(1) $$\phi_{ij}^{l/r}(t)=I_{ij}^{l/r}z_{ij}^{l/r}(t),$$

where *I*^*l/r*^_*ij*_ is the monocular input image and *z*^*l/r*^_*ij*_ (*t*) is a habituative gate that realizes an automatic gain control term simulating negative feedback mediated by Ca^2+^ ions, among others. It is defined as follows:

(2) $$\frac{dz_{ij}^{l/r}}{dt}=\left(B_{Z}-z_{ij}^{l/r}\right)-z_{ij}^{l/r}\left(C_{I}I_{ij}^{l/r}+C_{I^{*}}I^{*}\right),$$

where *B*_*Z*_ = 5 is the asymptote to which *z*^*l/r*^_*ij*_ (*t*) accumulates, or recovers, in the absence of input, and the term *z*^*l/r*^_*ij*_ (*C*_*I*_ *I*^*l/r*^_*ij*_ + *C*_I^*^_ I^*^) describes the inactivation of *z*^*l/r*^_*ij*_ by the present input, *I*^*l/r*^_*ij*_, and by a spatial average, I^*^, of all the inputs that approximates the effect of recent image scanning by sequences of eye movements. Parameters *C*_*I*_ = 2, and *C*_I^*^_ = 6. Solving Equations (1, 2) at equilibrium yields the equilibrium potential:

(3) $$\phi_{ij}^{l/r}=\frac{B_{Z}I_{ij}^{l/r}}{1+C_{I}I_{ij}^{l/r}+C_{I^{*}}I^{*}}.$$

In the simulations, I^*^ = 0.5 best approximates the effect of recent image scans.

The inner segment of the photoreceptor receives the signal ϕ^*l/r*^_*ij*_ from the outer segment and gets feedback *H*^*l/r*^_*ij*_ from the horizontal cells (HC) at position (*i, j*). HC modulation of the output of the inner segment of the photoreceptor is modeled by:

(4) $$\Phi_{ij}^{l/r}=\frac{\phi_{ij}^{l/r}}{B_{h}e^{H_{ij}^{l/r}}(B_{s}-\phi_{ij}^{l/r})+1},$$

where *B*_*h*_ = 0.05 is a small constant, and *B*_*s*_ = *B*_*z*_ / *C*_*I*_ = 2.5. This constant value of *B*_*s*_ ensures that perfect shifts (viz., adaptation) of the log(*I*^*l/r*^_*ij*_) − Φ^*l/r*^_*ij*_ curve occur as *H*^*l/r*^_*ij*_ is varied. For more details, see Grossberg and Hong (2006). Many increasing functions of *H*^*l/r*^_*ij*_ will generate the shift property of Φ^*l/r*^_*ij*_ as a function of log (*I*^*l/r*^_*ij*_). Function *f*(*H*_*ij*_) = *B*_*h*_ *e*^*H*^*l/r*^_*ij*_^ was chosen because *e*^*H*^*l/r*^_*ij*_^ makes the sensitivity curve shift in an accelerating manner with increasing *H*^*l/r*^_*ij*_, where *H*^*l/r*^_*ij*_ is the sigmoid output of the HC at (*i, j*) in response to its potential *h*^*l/r*^_*ij*_:

(5) $$H_{ij}^{l/r}=\frac{a_{H}{\left[h_{ij}^{l/r}\right]}^{2}}{b_{H}^{2}+{\left[h_{ij}^{l/r}\right]}^{2}},$$

where *a*_*H*_ = 6 and *b*_*H*_ = 0.1.

The potential of an HC connected to its neighbors through gap junctions is defined as follows.

(6) $$\frac{dh_{ij}^{l/r}}{dt}=-h_{ij}^{l/r}+\sum_{pq\in N_{ij}^{H}}\Psi_{pqij}^{l/r}\left(h_{pq}^{l/r}-h_{ij}^{l/r}\right)+\Phi_{ij}^{l/r},$$

where Ψ^*l/r*^_*pqij*_ is the permeability between cells at (*i, j*) and (*p, q*); namely:

(7) $$\Psi_{pqij}^{l/r}=\frac{-1}{1+\;exp\left[-\left(\left|\Phi_{ij}^{l/r}-\Phi_{pq}^{l/r}\right|-\beta_{p}\right)/\mu_{p}\right]}+1,$$

where β_*p*_ = 0.01, and μ_*p*_ = 0.002, and *N*^*H*^_*ij*_ is the neighborhood of cells to which the HC at position (*i, j*) is connected:

(8) $$N_{ij}^{H}=\left\{(p,q):\sqrt{{\left(p-i\right)}^{2}+{\left(q-j\right)}^{2}}\leq 13\right\}.$$

### 5.2. LGN polarity-sensitive ON and OFF cells

#### 5.2.1. Center-surround processing

The retinally adapted signal Φ^*l/r*^_*ij*_ is processed by on-center off-surround (ON cells) and off-center on-surround (OFF) cells that obey the membrane, or shunting, equations of neurophysiology. The activity *x*^*l/r*, +^_*ij*_ of the on-center off-surround (ON) network that receives input signals Φ^*l/r*^_*ij*_ (Equation 4) from the inner segment of the photoreceptors is defined as follows:

(9) $$\begin{array}{l} \frac{dx_{ij}^{l/r,\;+}}{dt}=-x_{ij}^{l/r,\;+}+\left(1-x_{ij}^{l/r,\;+}\right)\left(0.6\Phi_{ij}^{l/r}\right)\\ \;-\left(x_{ij}^{l/r,\;+}+1\right)E_{ij}^{l/r}+\Theta^{l/r,\;+}. \end{array}$$

In Equation (9), the term 0.6 Φ^*l/r*^_*ij*_ is the on-center input, *E*^*l/r*^_*ij*_ is the off-surround input, and Θ^*l/r*, +^ is the resting activity. The off-surround obeys:

(10) $$E_{ij}^{l/r}=\;\frac{0.6\left(\sum_{(p,q)\in \;N_{ij}^{E}}\Phi_{pq}^{l/r}E_{pqij}^{l/r}\right)}{\sum_{(p,q)\in \;N_{ij}^{E}}E_{pqij}^{l/r},}\;$$

where *N*^*E*^_*ij*_ is the off-surround neighborhood to which the cell at (*i, j*) is connected:

(11) $$N_{ij}^{E}=\left\{(p,q):\sqrt{{\left(p-i\right)}^{2}+{\left(q-j\right)}^{2}}\leq 6\right\},$$

and *E*^*l/r*^_*pqij*_ is the inhibitory off-surround kernel:

(12) $$E_{pqij}^{l/r}=\frac{0.6e^{\left(-\frac{{\left(p-i\right)}^{2}+{\left(q-j\right)}^{2}}{16}\right)}}{\sum_{(p,q)\in \;N_{ij}^{E}}e^{\left(-\frac{p^{2}+q^{2}}{16}\right)}},$$

which is normalized by the terms in the denominator. With this LGN ON-center/OFF-surround processing, the single and double-opponent LGN polarity-sensitive cells can be computed as follows.

#### 5.2.2. ON/OFF channels and double-opponent cells

As defined in Grossberg et al. (1995), the equilibrium, ON-cell activities of Equation (9) are thresholded to yield the output signals:

(13) $$x_{ij}^{l/r,\;+}={\left[\frac{\Theta^{l/r,\;+}+0.6\Phi_{ij}^{l/r}-E_{ij}^{l/r}}{1+0.6\Phi_{ij}^{l/r}+E_{ij}^{l/r}}\right]}^{+}.$$

The corresponding equilibrium outputs of the off-center on-surround (OFF) network are:

(14) $$x_{ij}^{l/r,\;-}={\left[\frac{\Theta^{l/r,\;-}+E_{ij}^{l/r}-0.6\Phi_{ij}^{l/r}}{1+0.6\Phi_{ij}^{l/r}+E_{ij}^{l/r}}\right]}^{+}.$$

By (14), the on-center and off-surround of an OFF cell is the off-surround and the on-center of the corresponding ON cell, respectively. The rest level parameters Θ^+^ and Θ^−^ were chosen with Θ^−^ > Θ^+^ — in particular, Θ^*l/r*, +^ = 1.5 and Θ^*l/r*, −^ = 4.5, which allows the OFF cells to be tonically active in the presence of uniform inputs, including in the dark. The inhibitory interactions that define the ON and OFF cells in Equations (13, 14) are computed across space among other ON and OFF cells, respectively. In contrast, the next processing stage of, double-opponent cells is defined by subtracting the ON and OFF cell output output signals at each position, and then thresholding the result:

***Double-opponent ON-cell:***

(15) $$X_{ij}^{l/r,\;+}={\left[x_{ij}^{l/r,\;+}-x_{ij}^{l/r,\;-}\right]}^{+}.$$

***Double-opponent OFF-cell:***

(16) $$X_{ij}^{l/r,\;-}={\left[x_{ij}^{l/r,\;-}-x_{ij}^{l/r,\;+}\right]}^{+}.$$

### 5.3. Boundary processing

#### 5.3.1. Simple cells

The simple cell activities *T*^*l/r*^_*ij*θ_ in model cortical area V1 receive their inputs from double-opponent LGN cells and are computed as in Raizada and Grossberg (2003). At each position (*i, j*), and for each of the four orientations θ = {0°, 45°, 90°, 135°}, a Difference-of-Offset-Gaussian (DOOG) kernel was used to compute each simple cell's orientationally-tuned ON and OFF subregions. In response to an oriented contrast edge in an input image, a suitably oriented simple cell of correct polarity will have its ON subfield stimulated by a luminance increment and its OFF subfield stimulated by a luminance decrement. The simple cell activity *T*^*l/r*^_*ij*θ_ for a given orientation θ, is the rectified sum of activities of each subfield, minus their difference:

(17) $$T_{ij\theta }^{l/r}=\vartheta {\left[U_{ij\theta }^{l/r}+V_{ij\theta }^{l/r}-\left|U_{ij\theta }^{l/r}-V_{ij\theta }^{l/r}\right|\right]}^{+},$$

where ϑ = 6, and the term *U*^*l/r*^_*ij*θ_ and *V*^*l/r*^_*ij*θ_ in Equation (17) represent the ON and OFF subregions, respectively:

(18) $$U_{ij\theta }^{l/r}=\sum_{mn}\left({\left[X_{mn}^{l/r,\;+}\right]}^{+}-{\left[X_{mn}^{l/r,\;-}\right]}^{+}\right){\left[D_{mnij\theta }^{l/r}\right]}^{+}$$

and

(19) $$V_{ij\theta }^{l/r}=\sum_{mn}\left({\left[X_{mn}^{l/r,\;-}\right]}^{+}-{\left[X_{mn}^{l/r,\;+}\right]}^{+}\right){\left[-D_{mnij\theta }^{l/r}\right]}^{+},$$

and *D*^*l/r*^_*mnij*θ_ is the DOOG kernel:

(20) $$D_{mnij\theta }^{l/r}=\frac{1}{2\pi \sigma_{D}^{2}}\left[\begin{array}{c} \exp\left(-\frac{{\left(m-i+\delta \cos\theta \right)}^{2}+{\left(n-j+\delta \sin\theta \right)}^{2}}{2\sigma_{D}^{2}}\right)\\ -\\ \exp\left(-\frac{{\left(m-i-\delta \cos\theta \right)}^{2}+{\left(n-j-\delta \sin\theta \right)}^{2}}{2\sigma_{D}^{2}}\right) \end{array}\right]$$

in which σ_*D*_ = 0.5 is the standard deviation of the kernel width.

#### 5.3.2. Complex cells

The model boundary is not used to simulate any polarity-specific properties. Thus, for simplicity, the simple cell responses are pooled across all four orientations to define the complex cell activities and output signals:

(21) $$Z_{ij}^{l/r}=0.25\sum_{\theta }T_{ij\;\theta }^{l/r}$$

#### 5.3.3. Monocular retinotopic boundaries

The monocular retinotopic boundary activities *R*^*l/r*^_*ij*_ (Figure 2) obey:

(22) $$\begin{array}{l} \frac{dR_{ij}^{l/r}}{dt}=-a_{R}R_{ij}^{l/r}+\left(b_{R}-R_{ij}^{l/r}\right)\left(Z_{ij}^{l/r}+c\sum_{klij}h\left(G_{klij}^{R^{l/r}}\right)E_{klij}^{IR}\right)\\ \;-\left(R_{ij}^{l/r}+d_{R}\right)\left(\sum_{pq}Z_{pq}^{l/r}+d\sum_{klij}h\left(G_{klij}^{R^{l/r}}\right)E_{klij}^{IR}\right), \end{array}$$

where the decay rate α_*R*_ = 5, the shunting excitatory saturation activity *b*_*R*_ = 10, and the shunting inhibitory saturation activity *d*_*R*_ = 2. A bottom-up on-center *Z*^*l/r*^_*ij*_ off-surround ∑_*pq*_*Z*^*l/r*^_*pq*_ network of inputs come from complex cell outputs *Z*^*l/r*^_*ij*_. Retinotopic monocular boundaries also receive top-down on-center off-surround signals ∑_*klij*_*h*(*G*^*R*^*l/r*^^_*klij*_) *E*^*IR*^_*klij*_ from invariant, or head-centered, monocular boundaries that are first transformed by gain fields. Functions *G*^*R*^*l/r*^^_*klij*_ are the top-down gain field output signals from position (*k, l*) to (*i, j*), and *E*^*IR*^_*klij*_ are the top-down connection weights from this gain field to the retinotopic boundary cells. These gain field functions and weights are defined in Equations (28–32). The feedback signal function *h* is threshold-linear:

(23) $$h(a)={\left[a-0.2\right]}^{+}.$$

These top-down gain field signals are multiplied in Equation (22) by excitatory and inhibitory gains *c* = 10 and *d* = 2, respectively.

#### 5.3.4. Invariant monocular boundaries

The invariant monocular boundary activities *B*^*l/r*^_*ij*_ receive bottom-up inputs via gain fields *G*^*R*^*l/r*^^_*klij*_ that transform the retinotopic monocular boundaries into invariant monocular boundaries (Figure 3). Before an eye movement occurs, the dark-light monocular invariant boundary activity is defined to equal the corresponding retinotopic monocular boundary activity:

(24) $$B_{ij}^{l/r,\;+}=R_{ij}^{l/r},$$

and the light-dark monocular invariant boundary activity is defined as

(25) $$B_{ij}^{l/r,\;-}=\{\begin{array}{l} {\left[1-B_{ij}^{l/r,\;+}\right]}^{+}\text{ if }B_{ij}^{l/r,\;+}\neq 0\\ 0\text{ otherwise}. \end{array}$$

As eye movements occur, the invariant monocular boundaries receive retinotopic monocular boundary inputs (Equation 22) through the gain fields *G*^*R*^*l/r*^^_*klij*_ described in Equations (28–32). Their left (L) *B*^*l*, +/−^_*ij*_ and right (R) *B*^*r*, +/−^_*ij*_ activities are defined as follows:

(26) $$\begin{array}{l} \frac{dB_{ij}^{l/r,+/-}}{dt}=-a_{b}B_{ij}^{l/r,+/-}+\left(1-B_{ij}^{l/r,+/-}\right)(f\left(B_{ij}^{l/r,+/-}\right)\\ \;+p_{b}\sum_{klij}h\left(G_{klij}^{R^{l/r}}\right)E_{klij}^{IB}+\lambda h\left(B_{ij}^{b,+/-}\right))\\ \;-B_{ij}^{l/r,+/-}\sum_{kl}(f\left(B_{kl}^{l/r,+/-}\right)\text{​}+\text{​}q_{b}\sum_{klij}h\left(G_{klij}^{R^{l/r}}\right)\text{​}E_{klij}^{IB}\\ \;+h\left(B_{kl}^{b,+/-}\right)), \end{array}$$

where *a*_*b*_ = 20 is the decay rate, and

(27) $$f(a)=\frac{a^{2}}{4+2a^{2}}$$

is the feedback sigmoid signal function that transforms the activities of the invariant monocular boundaries into a recurrent on-center off-surround network of feedback signals that maintain the persistent activity of the invariant boundaries in the network. Parameters *p*_*b*_ = 16 and *q*_*b*_ = 16 are excitatory and inhibitory gains that multiply the bottom-up excitatory and inhibitory signals, respectively, from the gain fields. Invariant monocular boundaries receive the same bottom-up excitatory and inhibitory signals ∑_*klij*_*h*(*G*^*R*^*l/r*^^_*klij*_) *E*^*IB*^_*klij*_ from retinotopic monocular boundaries that are first transformed by gain fields. Functions *G*^*R*^*l/r*^^_*klij*_ are the bottom-up gain field output signals from position (*k, l*) to (*i, j*), and *E*^*IB*^_*klij*_ are the bottom-up connection weights from this gain field to the retinotopic boundary cells. These gain field functions and weights are defined in (Equations 28–32). Parameter λ = 1.5 is a gain constant that multiplies the excitatory feedback signal *h*(*B*^*b*,+/−^_*ij*_) from the invariant binocular boundary *B*^*b*,+/−^_*ij*_ (Equation 33). The inhibitory feedback signal *h*(*B*^*b*,+/−^_*ij*_) has a gain of 1. Signal function *h*is the threshold-linear function defined in Equation (23).

#### 5.3.5. Boundary gain fields

Boundary gain field activities *G*^*R*^*l/r*^^_*klij*_ receive inputs from retinotopic monocular boundary signals *R*^*l/r*^_*ij*_ (Equation 22), predictive eye position signals *P*_*ij*_ (Equation 66), and invariant monocular boundary signals *B*^*l/r*,+/−^_*ij*_ (Equation 26 and Figure 3) in order to activate and maintain the invariant monocular boundaries *B*^*l/r*,+/−^_*ij*_ (Equation 26):

(28) $$\begin{array}{l} \frac{dG_{klij}^{R^{l/r}}}{dt}=\left(1-G_{klij}^{R^{l/r}}\right)\\ \left(\sum_{ij}R_{ij}^{l/r}E_{klij}^{RI}+\sum_{ij}P_{ij}E_{klij}^{PI}+\sum_{ij}B_{ij}^{l/r,+/-}E_{klij}^{BI}\right)\\ -(G_{klij}^{R^{l/r}}+0.15)\sum_{klij}G_{klij}^{R^{l/r}}. \end{array}$$

Gaussian kernels *E*^*RI*^_*klij*_, *E*^*PI*^_*klij*_, and *E*^*BI*^_*klij*_ represent the gain field weights from each of these input sources:

(29) $$E_{klij}^{RI}\;=\exp\left(-\frac{{\left(k-i\right)}^{2}+{\left(l-j\right)}^{2}}{2\sigma_{G_{R}^{R}}^{2}}\right);\sigma_{G_{R}^{RI}}=2$$

(30) $$E_{klij}^{PI}=\exp\left(-\frac{{\left(k-i\right)}^{2}+{\left(l-j\right)}^{2}}{2\sigma_{G_{R}^{P}}^{2}}\right);\sigma_{G_{R}^{PI}}=2$$

(31) $$E_{klij}^{BI}\;=\exp\left(-\frac{{\left(k-i\right)}^{2}+{\left(l-j\right)}^{2}}{2\sigma_{G_{R}^{B}}^{2}}\right);\sigma_{G_{R}^{BI}}=3.5$$

The top-down and bottom-up gain field weights are the same. Separate copies of these weights are defined for conceptual clarity:

(32) $$E_{klij}^{BI}=E_{klij}^{IB};\;E_{klij}^{PI}=E_{klij}^{IP};\;E_{klij}^{RI}=E_{klij}^{IR}$$

#### 5.3.6. Invariant binocular boundaries

The model considers how a 2D planar surface that is viewed in 3D is binocularly fused and how its 3D boundaries and surfaces are maintained during eye movements. It assumes a fixed, but otherwise arbitrary, binocular disparity of the left and right eye monocular boundaries corresponding to the object's image contours. The output signals *B*^*l/r*^_*ij*_ from the left and the right invariant monocular boundaries (Figure 3 and Equation 26) are binocularly fused as follows to create the invariant binocular boundary activities *B*^*b*^_*ij*_:

(33) $$\begin{array}{l} \frac{dB_{ij}^{b,+/-}}{dt}=-\gamma_{1}B_{ij}^{b,+/-}+\left(1-B_{ij}^{b,+/-}\right)\\ \;\left({\left[B_{(i\;+\;s)j}^{l,+/-}-\kappa \right]}^{+}+{\left[B_{(i\;-\;s)j}^{r,+/-}-\kappa \right]}^{+}\right)\\ \;+\left(1+3.2\sum_{klij}h\left(G_{klij}^{C}\right)J_{klij}^{CB}\right)-\alpha ({\left[O_{ij}^{l,+/-}\right]}^{+}\\ \;+\left[O_{ij}^{l,-/+}\right]+{\left[O_{ij}^{r,+/-}\right]}^{+}+{\left[O_{ij}^{r,-/+}\right]}^{+}), \end{array}$$

where γ_1_ = 0.1 is the rate of decay of the membrane potential. In Equation (33), the binocular disparity is assumed to cause allelotropically shifted monocular boundary signals *B*^*l*,+/−^_(*i* + *s*)*j*_ and *B*^*r*,+/−^_(*i* − *s*)*j*_, with shift *s*, which are binocularly fused via the sum [*B*^*l*,+/−^_(*i* + *s*)*j*_ − κ]^+^ + [*B*^*r*,+/−^_(*i* − *s*)*j*_ − κ]^+^, where κ = 0.4 is the boundary signal threshold. The selectivity of binocular fusion is achieved by balancing these excitatory terms against the sum of inhibitory signals α([*O*^*l*,+/−^_*ij*_]^+^ + [*O*^*l*,−/+^_*ij*_] + [*O*^*r*,+/−^_*ij*_]^+^ + [O^*r*,−/+^_*ij*_]^+^), where α = 7.2 is the inhibitory gain. Together, these balanced excitatory and inhibitory terms help to realize the *obligate property* (Poggio, 1991; Grossberg and Howe, 2003), whereby these binocular cells respond only to left and right eye inputs of approximately equal size, one of the important prerequisites for solving the *correspondence problem* of binocular vision (Howard and Rogers, 1995, pp. 42, 43).

The left *O*^*l*,+/−^_*ij*_ and right *O*^*r*,+/−^_*ij*_ inhibitory interneuron cell activities that ensure the obligate property are defined by:

(34) $$\begin{array}{l} \frac{dO_{ij}^{l,+/-}}{dt}=-\gamma_{2}O_{ij}^{l,+/-}+{\left[B_{(i\;+\;s)j}^{l,+/-}-\kappa \right]}^{+}\\ \;-\beta \left({\left[O_{ij}^{r,+/-}\right]}^{+}+{\left[O_{ij}^{r,-/+}\right]}^{+}+{\left[O_{ij}^{l,-/+}\right]}^{+}\right) \end{array}$$

and

(35) $$\begin{array}{l} \frac{dO_{ij}^{r,+/-}}{dt}=-\gamma_{2}O_{ij}^{r,+/-}+{\left[B_{(i\;-\;s)j}^{r,+/-}-\kappa \right]}^{+}\\ \;-\beta \left({\left[O_{ij}^{l,+/-}\right]}^{+}+{\left[O_{ij}^{l,-/+}\right]}^{+}+{\left[O_{ij}^{r,-/+}\right]}^{+}\right), \end{array}$$

where the decay rate γ_2_ = 4.5; [*B*^*l/r*,+/−^_(*i* + *s*)*j*_ − κ]^+^ are the excitatory signals from the monocular invariant boundaries that drive the inhibitory interneurons; and β = 4 is the gain of the recurrent inhibitory signals β([*O*^*r*,+/−^_*ij*_]^+^ + [*O*^*r*,−/+^_*ij*_]^+^ + [*O*^*l*,−/+^_*ij*_]^+^) among the inhibitory interneurons that are needed to ensure the obligate property (Grossberg and Howe, 2003). In Equations (33–35), the subscript *s* denotes the allelotropic, or positional, shift between the left and the right eyes that depends on the disparity to which the model neurons are tuned. In the simulations, results are shown for an allelotropic shift of *s* = +3^*o*^ to illustrate neurons that are tuned to a far disparity. The simulations also work for other binocular disparities and the allelotropic shifts that they induce. The obligate cell theorem from Grossberg and Howe (2003) was used to solve Equations 33–35 at equilibrium to speed up the simulations.

The invariant binocular boundaries in Equation (33) also receive feedback ∑_*klij*_*h*(*G*^*C*^_*klij*_) *J*^*CB*^_*klij*_ from the surface contour signals (Equation 45) that are generated from filled-in surfaces to their inducing boundaries. These surface contour signals enhance the corresponding closed boundaries, a crucial step in figure-ground separation whereby partially occluded object surfaces are separated in depth (Grossberg, 1994; Kelly and Grossberg, 2000). Since the fused binocular boundary is invariant, and thus computed in head-centered coordinates, but the surface contour is computed in retinotopic coordinates, the feedback from the surface contour is mediated through a gain field *G*^*C*^ to execute this coordinate change (Figure 4). The activity of the surface contour gain field *G*^*C*^ and the gain field kernel *J*^*CB*^ are defined in Equations (48, 49).

### 5.4. Surface processing

#### 5.4.1. Monocular retinotopic surface capture and filling-in

The monocular retinotopic surface filling-in activities *S*^*l/r*,+/−^_*ij*_ are computed from the brightness information that is driven by monocular retinotopic double-opponent ON and OFF cell activities *X*^*l/r*,+/−^_*ij*_ (Figure 2 and Equations 15, 16):

(36) $$\begin{array}{l} \frac{dS_{ij}^{l/r,+/-}}{dt}=-40S_{ij}^{l/r,+/-}+\sum_{pq\;\in \;N_{ij}}P_{pqij}^{l/r}\left(S_{pq}^{l/r,+/-}-S_{ij}^{l/r,+/-}\right)\\ \;+X_{ij}^{l/r,+/-}. \end{array}$$

The activities *S*^*l/r*,+/−^_*ij*_ diffuse via nearest-neighbor interactions via term ∑_*pq* ∈ *N*_*ij*__ *P*^*l/r*^_*pqij*_ (*S*^*l/r*,+/−^_*pq*_ − *S*^*l/r*,+/−^_*ij*_), where *N*_*ij*_ is the set of nearest neighbors around cell (*i, j*), and the permeability coefficients

(37) $$P_{pqij}^{l/r}=\frac{10^{4}}{0.01+20\left(K_{pq}^{b,+/-}+K_{ij}^{b,+/-}\right)}$$

are determined by binocular boundary gating signals *K*^*b*,+/−^_*pq*_ and *K*^*b*,+/−^_*ij*_ at positions (*p, q*) and (*i, j*), respectively. Since the binocular boundaries are computed in head-centered co-ordinates, whereas the monocular surfaces are computed in retinotopic coordinates, the boundary gating signals need to also be computed in retinotopic coordinates. This is accomplished by converting the binocular boundaries into retinotopic coordinates (Figure 4) using a predictive gain field:

(38) $$K_{ij}^{b,+/-}=\sum_{kl}h\left(G_{klij}^{S,+/-}\right)Q_{klij}^{BS}$$

that is defined in Equations (42–44).

#### 5.4.2. Binocular retinotopic surface capture and filling in

The binocular surface representations are preserved during eye movements, even though they are computed in retinotopic coordinates, due to the action of predictive gain fields that control the binocular filling-in process. In particular, the retinotopic surface filling-in activities *S*^*b*,+/−^_*ij*_ are activated by the rectified sum [*S*^*l*,+/−^_*ij*_]^+^ + [*S*^*r*,+/−^_*ij*_]^+^ of the monocular retinotopic surface activities captured by the invariant binocular boundary (Equation 36) corresponding to the same retinotopic position (*i, j*):

(39) $$\begin{array}{l} \frac{dS_{ij}^{b,+/-}}{dt}=-28S_{ij}^{b,+/-}+\sum_{pq\;\in \;N_{ij}}N_{pqij}\left(S_{pq}^{b,+/-}-S_{ij}^{b,+/-}\right)\\ \;+{\left[S_{ij}^{l,+/-}\right]}^{+}\text{​​}+\text{​​}{\left[S_{ij}^{r,+/-}\right]}^{+}\text{​}+\text{​}9\sum_{kl}h\left(G_{klij}^{A}\right)M_{klij}^{IS} \end{array}$$

The binocular surface activities undergo diffusion $\sum_{pq\;\in \;N_{ij}}N_{pqij}\left(S_{pq}^{b,+/-}-S_{ij}^{b,+/-}\right)$ in response to these input signals. The diffusion takes place among their nearest-neighbor cells *N*_*ij*_, whose permeabilities

(40) $$N_{pqij}=\frac{10^{4}}{0.01+20\left(K_{pq}^{b,+/-}+K_{ij}^{b,+/-}\right)}$$

are determined by binocular boundary gating signals *K*^*b*,+/−^_*pq*_ and *K*^*b*,+/−^_*ij*_ at positions (*p, q*) and (*i, j*), respectively. Similar to the monocular surfaces, binocular surfaces are as well computed in retinotopic coordinates. However, the binocular boundaries are computed in head-centered co-ordinates and thus the boundary gating signals need to also be computed in retinotopic coordinates. This is accomplished by converting the binocular boundaries into retinotopic coordinates (Figure 4) using a predictive gain field. The retinotopic boundary gating signals *K*^*b*,+/−^_*ij*_ were defined earlier in Equation (38). The gain fields for accomplishing this conversion are defined in Equations (42–44).

The binocular surface representation also receives top-down excitatory feedback from spatial attention (Figure 4) to induce and maintain a surface-shroud resonance. Spatial attention is in head-centered coordinates, whereas the binocular surface representation is retinotopic. Hence the spatial attentional feedback ∑_*kl*_*h*(*G*^*A*^_*klij*_)*M*^*IS*^_*klij*_ in Equation (39) is also computed in retinotopic coordinates using the predictive gain field *G*^*A*^_*klij*_ that is defined by Equations (56–60).

*S*^*b*,+/−^_*ij*_ is the fused binocular surface representation that is maintained in retinotopic coordinates despite eye movements across the visual scene. These ON and OFF binocular FIDO activities are rectified and combined to yield the final binocular surface percept:

(41) $$S^{b}={\left[S^{b,+}\right]}^{+}+{\left[S^{b,-}\right]}^{+}$$

In the simulation results, *S*^*b*^ is shown as the final binocular surface percept. This rectified summation of the ON and OFF domains enables surface-shroud resonance by attracting spatial attention on both light and dark filled-in surfaces. However, all the different representations, not just of brightness information, but also of brightness and color in depth, can be held as separate representations. The ensemble of all such parallel representations is what is learned, recognized, and categorized as belonging to a particular object in the What stream.

#### 5.4.3. Surface gain fields

The gain fields that enable binocular invariant boundaries to gate binocular and monocular surface percepts are defined as follows. Surface gain fields receive inputs from binocular invariant boundaries and predictive eye position signals (Figure 4):

(42) $$\begin{array}{l} \frac{dG_{klij}^{S,+/-}}{dt}=\left(1-G_{klij}^{S,+/-}\right)\left(\sum_{ij}B_{ij}^{b,+/-}Q_{klij}^{BS}+\sum_{ij}P_{ij}Q_{klij}^{PS}\right)\\ \;-\left(G_{klij}^{S,+/-}+0.37\right)\sum_{klij}G_{klij}^{S,+/-} \end{array}$$

where *B*^*b*,+/−^_*ij*_ is the invariant binocular boundary activity defined in (Equation 33), and *P*_*ij*_ is the predictive eye position described in Equation (66). Gaussian kernels *Q*^*BS*^_*klij*_ and *Q*^*PS*^_*klij*_ multiply the invariant binocular boundary signals and the eye position signals, respectively:

(43) $$Q_{klij}^{PS}=\exp\left(-\frac{{\left(k-i\right)}^{2}+{\left(l-j\right)}^{2}}{2\sigma_{G_{S}^{PS}}^{2}}\right);\;\sigma_{G_{S}^{PS}}=1.2$$

(44) $$Q_{klij}^{BS}=\exp\left(-\frac{{\left(k-i\right)}^{2}+{\left(l-j\right)}^{2}}{2\sigma_{G_{S}^{BS}}^{2}}\right);\;\sigma_{G_{S}^{BS}}=1.4$$

#### 5.4.4. Surface contour activity

The binocular surface activities *S*^*b*^_*pq*_ (Equation 41) are contrast-enhanced by on-center off-surround output networks to generate surface contour signals that modulate the invariant binocular boundaries (Figure 3 and Equation 33) and, through them, the corresponding retinotopic boundaries (Equation 22). Surface contour signals (Figure 4) are also used to determine the predictive target position signal (Equation 66) that maintains the stability of boundaries, surfaces, and attentional shrouds in head-centered coordinates via gain fields (Figures 1, 3, 4), even before the next eye movement is made, and to generate this eye movement signal. Surface contour signals occur only at positions corresponding to the boundary contours of the surface. The contour signals *C*_*ij*_ obey:

(45) $$\begin{array}{l} C_{ij}={\left[\frac{\sum_{pq}S_{pq}^{b}\left(\Lambda_{pqij}^{+}-\Lambda_{pqij}^{-}\right)}{0.04+\sum_{pq}S_{pq}^{b}\left(\Lambda_{pqij}^{+}+\Lambda_{pqij}^{-}\right)}\right]}^{+}\\ \;+{\left[\frac{\sum_{pq}S_{pq}^{b}\left(\Lambda_{pqij}^{-}-\Lambda_{pqij}^{+}\right)}{0.04+\sum_{pq}S_{pq}^{b}\left(\Lambda_{pqij}^{+}+\Lambda_{pqij}^{-}\right)}\right]}^{+}, \end{array}$$

where Λ^+^_*pqij*_ and Λ^−^_*pqij*_ are the contrast-enhancing *S*^*b*^ on-center and off-surround kernels, respectively:

(46) $$\Lambda_{pqij}^{+}=\frac{1}{3.61}\exp\left(-\frac{{\left(p-i\right)}^{2}+{\left(q-j\right)}^{2}}{2\sigma_{\Lambda^{+}}^{2}}\right);\sigma_{\Lambda^{+}}=0.5$$

(47) $$\Lambda_{pqij}^{-}=\frac{1}{12.27}\exp\left(-\frac{{\left(p-i\right)}^{2}+{\left(q-j\right)}^{2}}{2\sigma_{\Lambda^{+}}^{2}}\right);\sigma_{\Lambda^{-}}=2$$

#### 5.4.5. Gain fields from surface contour to invariant binocular boundary

Since the surface contour is in retinotopic coordinates and the fused binocular boundary that it modulates is in head-centered coordinates, a gain field *G*^*C*^_*klij*_ transforms the input from surface contour to binocular boundary (Figure 4):

(48) $$\begin{array}{l} \frac{dG_{klij}^{C}}{dt}=\left(1.8-G_{klij}^{C}\right)\left(\sum_{ij}C_{ij}J_{klij}^{CB}+\sum_{ij}P_{ij}J_{klij}^{PB}+\right)\\ \;-\left(G_{klij}^{C}+0.7\right)\sum_{klij}G_{klij}^{C}, \end{array}$$

where *C*_*ij*_ is the surface contour activity defined in Equation (45), and *P*_*ij*_ is the predictive target position signal described in Equation (66). Terms *J*^*CB*^_*klij*_, and *J*^*PB*^_*klij*_ in Equation (48) represent the Gaussian gain field kernels that transform the surface contour and the target position signals, respectively:

(49) $$J_{klij}^{CB}=\exp\left(-\frac{{\left(k-i\right)}^{2}+{\left(l-j\right)}^{2}}{2\sigma_{G_{C}^{CB}}^{2}}\right);\sigma_{G_{C}^{CB}}=2.6$$

(50) $$J_{klij}^{PB}=\exp\left(-\frac{{\left(k-i\right)}^{2}+{\left(l-j\right)}^{2}}{2\sigma_{G_{C}^{PB}}^{2}}\right);\sigma_{G_{C}^{CB}}=1.2$$

### 5.5. Spatial shrouds

#### 5.5.1. Spatial attention activity

The spatial attention cell activities *A*_*ij*_ that support attentional shrouds obey:

(51) $$\begin{array}{l} \frac{1}{10}\frac{dA_{ij}}{dt}=-0.2A_{ij}+\left(2-A_{ij}\right)\left(A_{ij}^{I}+\sum_{mn}g(A_{mn})\Omega_{mnij}^{+}\right)y_{ij}^{A}\\ \;-A_{ij}\left(\sum_{mn}\left(A_{mn}^{I}+g\left(A_{mn}\right)\Omega_{mnij}\right)+C_{RESET}y^{c}\right). \end{array}$$

These cell activities receive bottom-up excitatory inputs *A*^*I*^_*ij*_ from the corresponding attention interneurons (see Equation 55). They also receive recurrent on-center signals ∑_*mn*_*g*(*A*_*mn*_) Ω^+^_*mnij*_ and off-surround signals *g*(*A*_*mn*_)Ω^−^_*mnij*_ from other spatial attention cells, where *g* is a sigmoid signal function that converts cell activities into output signals:

(52) $$g(a)=\frac{7}{1+e^{-25a\;+\;11}}.$$

Kernels Ω^+^_*mnij*_, and Ω^−^_*mnij*_ are the on-center and off-surround Gaussian weights, respectively, from position (*m, n*) to position (*i, j*):

(53) $$\Omega_{mnij}^{+}=0.04\exp\left(-\frac{{\left(m-i\right)}^{2}+{\left(n-j\right)}^{2}}{2\sigma_{\Omega^{+}}^{2}}\right);\sigma_{\Omega^{+}}=0.5$$

(54) $$\Omega_{mnij}^{-}=2.2\exp\left(-\frac{{\left(m-i\right)}^{2}+{\left(n-j\right)}^{2}}{2\sigma_{\Omega^{-}}^{2}}\right);\sigma_{\Omega^{-}}=100$$

The excitatory inputs and recurrent signals in Equation (51) are multiplied by habituative attentional transmitter gates *y*^*A*^_*ij*_ (Equation 61) that enable inhibition-of-return (IOR). The system also receives a parietal reset signal *C*_*RESET*_ (Equation 62) that inhibits the currently active shroud. The reset signal *C*_*RESET*_ is multiplied by a habituative transmitter gate *y*^*C*^ (Equation 63) which ensures that the net reset signal *C*_*RESET*_ *y*^*C*^ is transient.

#### 5.5.2. Attentional interneuron cell activity

Attentional interneuronal activities *A*^*I*^_*ij*_ input to the spatial attention cell activities in Equation (51), receive reciprocal top-down feedback from the spatial attention cells (Figures 4, 5), and are themselves activated by bottom-up signals from the binocular filled-in surfaces (Equation 41) to form surface-shroud resonances:

(55) $$\frac{dA_{ij}^{I}}{dt}=-0.9A_{ij}^{I}+1.2\sum_{kl}h\text{​}\left(G_{klij}^{A}\right)M_{klij}^{IA}+g\text{​}\left(A_{ij}\right).$$

Because the binocular filled-in surfaces are computed in retinotopic coordinates, whereas the attentional shrouds are computed in head-center coordinates, gain fields are needed to transform their inputs between them. In Equation (55), ∑_*kl*_*h*(*G*^*A*^_*klij*_)*Q*^*IA*^_*klij*_ is the bottom-up input from the spatial attention gain fields.

#### 5.5.3. Gain fields for spatial attentional shrouds

The gain fields *G*^*A*^_*klij*_ from binocular surface to attentional interneuron (Figures 4, 5) obey:

(56) $$\begin{array}{l} \frac{dG_{klij}^{A}}{dt}=\left(1-G_{klij}^{A}\right)\left(\sum_{ij}S_{ij}^{b}M_{klij}^{SI}+\sum_{ij}P_{ij}M_{klij}^{PI}+\sum_{ij}A_{ij}^{I}M_{klij}^{AI}\right)\\ \;-\left(G_{klij}^{A}+0.37\right)\sum_{klij}G_{klij}^{A}, \end{array}$$

where *S*^*b*^_*ij*_ is the binocular surface representation (Equation 41), *P*_*ij*_ is the target position signal (Equation 66), and *A*^*I*^_*ij*_ is the attentional interneuronal activity (Equation 55). The Gaussian gain field kernels *M*^*SI*^_*klij*_, *M*^*PI*^_*klij*_, *M*^*AI*^_*klij*_ obey:

(57) $$M_{klij}^{SI}=\exp\left(-\frac{{\left(k-i\right)}^{2}+{\left(l-j\right)}^{2}}{2\sigma_{G_{A}^{SI}}^{2}}\right);\sigma_{G_{A}^{SI}}=3.2$$

(58) $$M_{klij}^{PI}=\exp\left(-\frac{{\left(k-i\right)}^{2}+{\left(l-j\right)}^{2}}{2\sigma_{G_{A}^{PI}}^{2}}\right);\sigma_{G_{A}^{PI}}=1.3$$

(59) $$M_{klij}^{AI}=\exp\left(-\frac{{\left(k-i\right)}^{2}+{\left(l-j\right)}^{2}}{2\sigma_{G_{A}^{AI}}^{2}}\right);\sigma_{G_{A}^{AI}}=5$$

In the simulations, the top-down and bottom-up gain field weights are symmetrical:

(60) $$M_{klij}^{SI}=M_{klij}^{IS};M_{klij}^{PI}=M_{klij}^{IP};M_{klij}^{AI}=M_{klij}^{IA}$$

#### 5.5.4. Habituative attentional transmitter gates

The habituative attentional transmitter gate (Equation 51) obeys:

(61) $$\frac{dy_{ij}^{A}}{dt}=\eta_{A}\left(\left(1.5-y_{ij}^{A}\right)-10^{3}A_{ij}^{I}y_{ij}^{A}\right),$$

where η_*A*_ = 10^−5^ is a slow rate of decay, (1.5 − *y*^*A*^_*ij*_) says that the gate *y*^*A*^_*ij*_ passively accumulates to a maximal activity of 1.5, and −10^3^ *A*^*I*^_*ij*_ *y*^*A*^_*ij*_ describes the activity-dependent habituation of *y*^*A*^_*ij*_.

#### 5.5.5. Shroud-mediated parietal reset and habituation

The parietal reset neurons are tonically active and their activities are inhibited by inputs from all the active cells across the spatial attention map. Their activity is disinhibited when an attentional shroud collapses, and generates a transient activity burst that inhibits, and resets, the spatial attention map. This reset mechanism (Chang et al., 2014) obeys:

(62) $$C_{RESET}=10{\left[1-\varepsilon -\frac{\sum_{ij}g(A_{ij})}{100+\sum_{ij}g(A_{ij})}\right]}^{+},$$

where ε = 0.07 is a small threshold, *A*_*ij*_ (Equation 51) is the activity of spatial attention at position *(i, j)* and *g* is defined in Equation (52).

The reset habituative transmitter *y*^*C*^ that gates the parietal reset signal obeys:

(63) $$\frac{dy^{C}}{dt}=10\left(0.75\left(1.5-y^{C}\right)-4C_{RESET}y^{C}\right).$$

As in Equation (61), this habituative gate also consists of a passive accumulation term 0.75(1.5 − *y*^*C*^) and an activity-dependent habituation term −4*C*_*RESET*_ *y*^*C*^.

### 5.6. Eye signals

#### 5.6.1. Eye movement signals to salient features and inhibition of return

Surface contour cell activities (Equation 45) are contrast-enhanced using a recurrent on-center off-surround network to choose the activity *F*_*ij*_ of the most salient feature, and thus the target position *(i, j)* for the next saccadic eye movement. A movement habituative transmitter gate weakens this choice in an activity-dependent way, thereby providing an inhibition-of-return mechanism which ensures that the same target position is not perseveratively chosen.

Salient feature *F*_*ij*_ at position (*i, j*) obeys:

(64) $$\begin{array}{l} \frac{dF_{ij}}{dt}=-15F_{ij}+\left(2-F_{ij}\right)\left({\left[C_{ij}\right]}^{+}+250F_{ij}^{2}\right)y_{ij}^{F}\\ \;-0.04F_{ij}\sum_{ij}\left({\left[C_{ij}\right]}^{+}+F_{ij}^{2}\right), \end{array}$$

where *C*_*ij*_ is the surface contour activity (Equation 45), and *y*^*F*^_*ij*_ is the movement habituative gate::

(65) $$\frac{dy_{ij}^{F}}{dt}=\eta_{F}\left((2-10^{5}y_{ij}^{F}\left({\left[C_{ij}\right]}^{+}+250F_{ij}^{2}\right)\right),$$

where η_*F*_ = 10^−4^ is rate of decay. Note that this rate of decay is an order of magnitude larger than η_*A*_, the rate of habituative decay for the spatial shrouds (Equation 61). Thus, the attentional shroud collapses much slower than inhibition-of-return of individual saccades that search the corresponding object (Chang et al., 2014). This rate difference enables multiple saccades within the attended surface to be explored and to thereby trigger learning of view-specific categories that encode multiple views of the attended object.

#### 5.6.2. Target position signal

The target position signal at (*i, j*) obeys:

(66) $$P_{ij}=\{\begin{array}{l} 1\text{ for }F_{ij}=\max_{ij}\left(F_{ij}\right)\;\forall \;(i,j)\\ 0\text{ otherwise}. \end{array}$$

This determines the next predictive eye position signal from the highest activity position, or salient feature, on the surface contour map (Equation 45). All the gain field cells for boundaries, surfaces, and spatial attention processing have access to this positional signal (cf. Pouget and Snyder, 2000).

## 6. Discussion

This article builds on the ARTSCAN and pARTSCAN models of how spatial attention in the Where stream modulates invariant object learning, recognition, and eye movement exploration of multiple object views in the What stream (Grossberg, 2007, 2009; Fazl et al., 2009; Cao et al., 2011; Foley et al., 2012; Chang et al., 2014). The 3D ARTSCAN model that is described herein extends these insights to explain how these processes can work in response to 3D objects and scenes. Together, these interacting processes model how mechanisms for maintaining stable binocular percepts of 3D objects are related to mechanisms for learning to invariantly categorize and recognize these objects.

A key insight of the current model concerns how predictive remapping through eye position-dependent gain fields maintains perceptual stability of binocularly fused images and scenes during saccadic eye movements. Additional processes of the 3D LAMINART model, a laminar cortical embodiment and further development of the FACADE model of 3D vision and figure-ground segregation (Grossberg, 1994, 1999; Kelly and Grossberg, 2000; Raizada and Grossberg, 2003; Grossberg and Swaminathan, 2004; Cao and Grossberg, 2005, 2012; Grossberg and Yazdanbakhsh, 2005; Fang and Grossberg, 2009), may be joined to the ARTSCAN model to clarify how more complex properties of 3D scenes than are simulated herein retain their perceptual stability under free viewing conditions.

### 6.1. FACADE and 3D ARTSCAN

FACADE theory proposes how visible 3D surfaces are captured by binocularly fused 3D boundaries. Surface capture is achieved when depth-selective filling-in of surface brightness and color is triggered by these boundaries through their function as *filling-in generators* (Grossberg, 1994). Boundaries also function as *filling-in barriers* that restrict filling-in within surface regions that the boundaries surround. The filled-in features can be derived either from bottom-up object brightness and color contrasts or from top-down attentional spotlights. An attentional spotlight can, for example, arise when top-down spatial attentional signals from parietal cortex modulate filled-in object surfaces in a depth-selective manner within visual cortical areas such as V4.

The 3D ARTSCAN model shows, in addition, how binocularly fused boundaries can use eye position-dependent gain fields to maintain fusion and an invariant head-centered representation during eye movements (Figure 3). These invariant boundaries can capture left and right eye monocular surface features in a depth-selective way (Figure 4). The captured monocular surfaces can, in turn, form and maintain binocular surfaces (Figure 4). An attended binocular surface is modulated by an attentional shroud, with gain fields again ensuring that the interactions are dimensionally consistent (Figure 4). Thus, during filling-in, surface contrasts are activated either bottom-up from the binocularly combined monocular surfaces after they are captured in depth by the binocular boundaries, or top-down from the surface's attentional shroud.

FACADE model retinal lightness adaptation, spatial contrast adaptation, and double opponent processing (Grossberg and Hong, 2006) are among the useful pre-processing stages that are incorporated in the 3D ARTSCAN model. The 3D ARTSCAN model does not, however, yet process chromatic natural scenes, such as in the aFILM simulations of anchoring (Hong and Grossberg, 2004; Grossberg and Hong, 2006); or orientationally-selective depth-selective boundary completion processes, such as in the 3D LAMINART model simulations of binocular stereograms (Fang and Grossberg, 2009), the LIGHTSHAFT model simulations of 3D shape-from-texture (Grossberg et al., 2007), and the FACADE model simulations of da Vinci stereopsis (Grossberg and McLoughlin, 1997; Cao and Grossberg, 2005, 2012); or moving-form-in-depth processes, such as in the 3D FORMOTION model simulations of coherent and incoherent plaid motion, speed perception, and the aperture problem (Chey et al., 1997, 1998), transformational apparent motion (Baloch and Grossberg, 1997), the chopsticks and rotating ellipse illusions (Berzhanskaya et al., 2007), and the barberpole illusion, line capture, and motion transparency (Grossberg et al., 2001). All of these other studies are computationally consistent with the 3D ARTSCAN model and hence their competences can be incorporated in future model extensions.

### 6.2. Attentional shrouds and surface-shroud resonances: seeing and knowing

The 3D ARTSCAN model also does not explicitly study invariant object category learning and recognition, although the concept of attentional shrouds in the ARTSCAN and pARTSCAN models, which plays a key role in modulating invariant category learning in those models, also clarifies in the current study how an object in depth maintains its perceptual stability and attentional focus during eye movements (Figures 1, 4).

The original use of the attentional shroud concept is closer to its perceptual role in 3D ARTSCAN than it is to its learned categorization role in ARTSCAN and pARTSCAN. In particular, the concept of an attentional shroud was introduced by Tyler and Kontsevich (1995) to clarify how spatial attention could morph itself to the shape of an object in depth, and how, in response to a transparent display, only one depth at a time might be perceived. Likova and Tyler (2003), also noted that “depth surface reconstruction is the key process in the accuracy of the interpolated profile from both depth and luminance signals” (see p. 2655), and thus that shroud formation involves surface fillng-in. However, they did not provide a design rationale or mechanistic explanation of these empirical facts.

The 3D ARTSCAN model does explain and simulate mechanistically how such depth-selective shrouds may form in the brain (Figure 4). Moreover, as noted above, the ARTSCAN family of models proposes how shrouds can form in response to either exogenously activated attention, via bottom-up inputs from objects in a scene, or endogenously activated attention, via a top-down route. In the 3D ARTSCAN model, once the attentional shroud fits itself to binocular surface input signals, the 3D surface-shroud resonance (Figures 4, 5) is the dynamical state corresponding to “paying spatial attention” to the object surface. Such a 3D surface-shroud resonance is a mechanistic revision and explanation of the proposal of Tyler and Kontsevich (1995, p. 138) that “stereoscopic-attentional process therefore would be much more valuable if it could be wrapped around the form of any spatial object, rather than being restricted to frontoparallel planes… more vivid representation of this process is to think of it as an attentional shroud, wrapping the dense locus of activated disparity detectors as a cloth wraps a structured object.” The 3D ARTSCAN model extends this view by proposing that it is the *3D surface-shroud resonance* which embodies a unified representation of consciously perceived object structure, not just the shroud taken alone, as in the Tyler and Kontsevich (1995) proposal. Boundary-category resonances and surface-category resonances are other aspects of object structure, whereby 3D boundary and surface representations interact reciprocally with their corresponding object category representations to invariantly categorize and recognize these object properties. Said more simply, these various resonances can synchronously represent seeing an object and knowing what it is.

### 6.3. Comparison with other models

To study object-based attention, LaBerge and Brown (1989) modeled attention as a gradient across the visual field with the peak at the expected target location. This gradient hypothesis could explain attention shifts better than a moving spotlight of attention, especially when spatial attention can form over more than one object. They also discussed how such a system could help in object recognition, especially in the identification of a visual shape in a cluttered scene. The model proved better than non-gradient based models of attention in explaining data on pre-cueing of locations in the visual field and of words.

Within the 3D ARTSCAN model, gradient properties can arise due to bottom-up properties of filling-in, the spatially distributed kernel that carries surface-to-shroud inputs, and the non-uniform distribution of shroud activity due to inhibition-of-return and activity-dependent habituation (Equations 51–66). Gradient properties can also be induced when a prefrontally-mediated top-down attentional spotlight, as modeled by Foley et al. (2012), remains on through time due to persistent volitional gain control (Brown et al., 2004; Grossberg, 2012, 2013) and combines with bottom-up shroud-maintaining mechanisms.

Logan (1996) integrated space-based and object-based approaches to visual attention by combining the COntour DEtector (CODE) theory of perceptual grouping by proximity (Van Oeffelen and Vos, 1982, 1983) with the Theory of Visual Attention (TVA) (Bundesen, 1990). In this unified Code Theory of Visual Attention (CTVA), CODE provides input to TVA, thereby accounting for spatially based between-object selection, while TVA converts the input to output, thereby accounting for feature- and category-based within-object selection. CODE clusters nearby items into emergent perceptual groupings that are both perceptual objects and regions of space, thereby integrating object-based and space-based approaches to attention. The theory assumes that attention chooses among perceptual objects by sampling the features that occur within an above-threshold region. The features of different items within this region are sampled with a probability that equals the area of the distribution of the item that falls within the region. This sampling probability is called the *feature catch*.

ARTSCAN also combines space-based and object-based visual attention. The space-based attention concerns how an object-fitting attentional shroud (cf. an “above-threshold region”) controls both the learning of invariant object categories and their recognition, including when recognition may break down due to the inability of a shroud to form around a target object, as is predicted to happen during perceptual crowding (Foley et al., 2012). At least three types of grouping occur in the ARTSCAN framework: The first concerns the kind of feature-based grouping of perceptual boundaries that explains Gestalt grouping laws (e.g., Grossberg and Pinna, 2012). The second concerns the surface grouping that occurs during a surface-shroud resonance. And the third concerns how these emergent boundary and surface representations are bound into view-specific categories, and how view-specific categories are, in turn, bound into invariant object categories. Object attention enters ARTSCAN in two ways: Adaptive Resonance Theory top-down expectations control the learning of ARTSCAN categories by focusing object attention upon predictive combinations of object features. Object attention also plays a key role in controlling a primed search for a desired object, as during a solution of the Where's Waldo problem, which is modeled by the ARTSCAN Search model (Chang et al., 2014). These various processes occur on multiple spatial and temporal scales, and clarify some of the complexities that occur when object and spatial attentional processes interact.

Visual attention and search models, such as Guided Search (Wolfe et al., 1989; Wolfe, 2007), and Saliency Map (Itti and Koch, 2001) models, have their genesis in Feature Integration Theory (Treisman and Gelade, 1980). In these models, the units are local features or positions. The models are thus *pixel-based*. The model mechanisms are based on competition between parallel visual representations, whereby a strong local salient feature wins and directs shifts in attention and eye movements to it (Deubel and Schneider, 1996; Deubel et al., 2002). In particular, in Saliency Map models, (e.g., Itti and Koch, 2001) different feature maps, such as brightness, orientation, color, or motion are computed in parallel visual representations. In each feature map, the strongest feature is selected by competition using an on-center, off-surround mechanism. The winning outputs of all these feature maps are then combined into a single map to build the saliency map. This saliency map predicts the probability with which a certain spatial positions will attract an observer's attention and eye movements.

Unlike pixel-based models, 3D ARTSCAN, as well as its ARTSCAN, pARTSCAN, dARTSCAN, and ARTSCAN Search variants, are *object-based* (Pylyshyn, 1989, 2001; Kahneman et al., 1992; Vergilino-Perez and Findlay, 2004) to enable the models to learn to attend, categorize, recognition, and search for objects in a scene. In these models, the competition for focusing attention, whether spatial (leading to a surface-shroud resonance) or object (leading to a feature-category resonance) is *regional* rather than local (Duncan, 1984).

The pre-processing of the 3D ARTSCAN model can be readily enhanced, as noted above, to include features such as color, orientation, and motion, as in the pixel-based models, but these features are bound into invariant binocular boundaries and retinotopic binocular surfaces which are the perceptual units that compete for spatial and object attention.

3D ARTSCAN can search a 3D scene to learn and recognize objects in it based on the salience of its boundary and surface properties, but it currently does so without accumulating evidence about contextual information. In contrast, in response to seeing a refrigerator and a stove, humans would expect to next see a sink more probably than a beach. 3D ARTSCAN does not learn such contextual expectations. In addition, 3D ARTSCAN, just like ARTSCAN and pARTSCAN before it, is devoted to *object*, rather than *scene*, perception, attention, learning, and recognition. 3D ARTSCAN is, however, one of a family of ART-based models (Carpenter and Grossberg, 1991, 1993) that do have these capabilities, and that can be combined in an enhanced future 3D ARTSCAN model.

For example, the ARTSCENE model (Grossberg and Huang, 2009) uses attentional shrouds to learn and recognize the gist of a scene as a large-scale texture category. ARTSCENE can also accumulate scenic evidence by using shrouds to iteratively focus attention on salient regions of the scene, and thereby learn texture categories at a finer scale, which can be combined by voting to improve scene recognition. However, ARTSCENE does not have a contextual memory of this accumulated scenic evidence through time.

Contextual cueing (e.g., Jiang and Chun, 2001; Olson and Chun, 2002) is modeled in the ARTSCENE Search model (Huang and Grossberg, 2010), which shows how spatial and object working memories can learn to accumulate and remember sequential contextual information to facilitate efficient search for an expected goal object, in the manner of the refrigerator/stove/sink example. In the ARTSCENE Search model, the object working memory involves perirhinal cortex interacting with prefrontal cortex, and the spatial working memory involves parahippocampal cortex, again interacting with prefrontal cortex. These brain regions also interact with inferotemporal and parietal cortices, respectively, among other brain areas, to determine where the eyes will look next. Thus, in ARTSCENE Search, each eye movement enables currently attended objects to be seen and recognized, while also triggering new category learning and working memory storage that can better predict goal objects in the future.

Another search variant that was mentioned above: the ARTSCAN Search model (Chang et al., 2014), uses pARTSCAN mechanisms to learn and recognize view- and positionally-invariant object categories using Where-to-What stream interactions. In addition, ARTSCAN Search can also search a scene for a valued goal object using What-to-Where stream interactions. Such a search may be activated by a top-down cognitive prime or motivational prime. The model hereby proposes a neurobiologically-grounded solution of the Where's Waldo problem.

### 6.4. Attentional gain control and normalization: a convergence across models

Recent models of attention have focused on studying the effects of attention on neuronal responses in visual cortical areas such as MT and V4 (e.g., Ghose, 2009; Lee and Maunsell, 2009; Reynolds and Heeger, 2009). These models explored how attention enhances processing of selected areas of the visual field, and concluded that divisive normalization using center-surround processing causes the effects of attention on V4 neurons. Top-down attentional priming had earlier been modeled in the FACADE, ART, and 3D LAMINART models using top-down, modulatory on-center, off-surround networks acting on cells that obey the membrane, or shunting, equations of neurophysiology (e.g., Carpenter and Grossberg, 1987, 1991, 1993; Gove et al., 1995; Grunewald and Grossberg, 1998; Grossberg et al., 2001; Berzhanskaya et al., 2007; Bhatt et al., 2007). In ART, such a top-down circuit for attention is called the ART Matching Rule. These ART results, in turn, built on the fact that cells which obey shunting dynamics in on-center off-surround anatomies automatically compute the property of divisive normalization. Grossberg (1973) provided an early mathematical proof of this normalization property, and Grossberg (1980) contained an early review.

More recently, there has been a convergence across models of how to mathematically instantiate the ART Matching Rule attentional circuit. For example, the “normalization model of attention” (Reynolds and Heeger, 2009) simulates several types of experiments on attention using the same equation for self-normalizing attention that the distributed ARTEXture (dARTEX) model (Bhatt et al., 2007, Equation A5) used to simulate human psychophysical data about Orientation-Based Texture Segmentation (OBTS, Ben-Shahar and Zucker, 2004). Whereas Reynolds and Heeger (2009) described an algebraic form-factor for attention, Bhatt et al. (2007) described and simulated the attentional dynamics whose steady state reduces to that form factor. Although the 3D ARTSCAN model uses shunting competitive dynamics to define its attentional modulation at multiple processing stages, it is difficult to summarize their net effect in a single steady-state equation due to the role of gain fields between surface and shroud representations to maintain perceptual stability during eye movements (see Equations 38–61).

### 6.5. Balancing object exploration vs. perseveration: inhibition-of-return

The brain can learn view-invariant object categories by exploring multiple salient features on each object. But why are not successive eye movement positions instead chosen randomly, thereby preventing efficient intra-object exploration? Indeed, psychophysical data support the idea that the eyes prefer to move within the same object for awhile (Theeuwes et al., 2010), rather than randomly. The stability of the surface-shroud resonance while the eyes explore an object's surface helps to explain how this happens. Such a resonance maintains spatial attention on a given object for awhile, while also enhancing the activity of the attended surface's surface contours. The most active position on a surface contour is chosen as the next saccadic target position on the attended object (Fazl et al., 2009), a transformation that is predicted to take place using cortical area V3A (Figure 1).

The brain must also solve the problem of not perseveratively choosing the same maximally activated position over and over again. Inhibition of return (IOR) is an important mechanism for any model of attention (List and Robertson, 2007), or, for that matter, any model of sequential performance. Perseverative performance of maximally active eye movement representations is prevented by their activity-dependent habituation as they are chosen to determine next eye movement target position (see Equations 64–66). This choice-dependent inhibitory feedback enables the 3D ARTSCAN model to choose the next most active position as the next saccadic target location. The combination of a self-normalizing activity map, selection of the maximal activity for the next output, and choice-dependent inhibitory feedback was introduced in Grossberg (1978a,b; see also Grossberg and Kuperstein, 1986) and has been used in many subsequent models, notably Koch and Ullman (1985).

### 6.6. Predictive remapping via eye command-mediated gain fields

Visual stability and object constancy requires the visual system to keep track of the spatiotopic or allocentric positions of several objects in a scene during saccades (Mathot and Theeuwes, 2010a,b). Retinotopic coordinates generate different representations of the same scene when it is viewed at different centers of gaze. This fact has led many investigators to conclude that retinotopic representations are predictively remapped by eye movement commands, with eye position-sensitive gain fields as a key remapping mechanism (Von Holst and Mittelstaedt, 1950; Von Helmholtz, 1867; Duhamel et al., 1992; Gottlieb et al., 1998; Tolias et al., 2001; Melcher, 2007, 2008, 2009; Saygin and Sereno, 2008; Mathot and Theeuwes, 2010a,b). Corollary discharges of outflow movement signals that act before the eyes stabilize on their next movement target are used to update the gain fields.

Several fMRI studies suggest that various visual representations in the Where, or dorsal, cortical stream that are sensitive to visual attention are computed in retinotopic coordinates. At least one area in anterior parietal cortex has been found using fMRI to be responsive to head-centered, or some sort of spatiotopic or absolute, coordinates (Sereno and Huang, 2006). Perisaccadic remapping of receptive fields has been reported in electrophysiological studies in frontal eye fields (Goldberg and Bruce, 1990), in parietal areas, including LIP (Andersen et al., 1990; Duhamel et al., 1992), and in V4 (Tolias et al., 2001). Interestingly, in these regions, after saccades, no new transient activity is caused when targets are attended (see Mathot and Theeuwes, 2010a for a review).

Psychophysical experiments have suggested that predictive remapping is mediated by predictive shifts of attention to the positions of intended targets. Cavanagh et al. (2010) called these shifts “attention pointers” (see Section 2.5). Predictive remapping of visual attention enables improved attentional performance that enhances perceptual processing at target positions and speeds up the eye movements to the new target's position (Rolfs et al., 2011). In the 3D ARTSCAN and related ARTSCAN models, the maximally active position on a surface contour is chosen as the next saccadic target position before the eye movement occurs, and causes a predictive updating of gain fields to maintain the stability of a currently active shroud and of the 3D surface percept during intra-object movements, and to facilitate the shift of spatial attention to a newly attended object (Sections 2.5 and 2.6). It therefore seems that the maximally active surface contour position, as described in the Fazl et al. (2009) ARTSCAN article, predicted key properties of the Cavanagh et al. (2010) attention pointer data. One way to test if this proposed connection is mechanistically sound is to link it to other ARTSCAN predictions. For example, are attention pointers computed in cortical area V3A (Figure 1), as is compatible with the data of Caplovitz and Tse (2007, p. 1179) showing “neurons within V3A… process continuously moving contour curvature as a trackable feature… not to solve the ‘ventral problem’ of determining object shape but in order to solve the ‘dorsal problem’ of what is going where”?

### 6.7. Retinotopic vs. spatiotopic representations

A recent behavioral study using fMRI in higher visual areas proposed that, in the dorsal visual stream and the intraparietal sulcus, all object locations are represented in retinotopic coordinates as their native coordinate system (Golomb and Kanwisher, 2012). These authors found little to no evidence of spatiotopic object position and suggested that a spatiotopic, or head-centered, ability to interact with objects in the world might be achieved by spatiotopic object positions that are “computed indirectly and continually reconstructed with each eye movement” (Golomb and Kanwisher, 2012, p. 2794), presumably using gain fields. One concern about an fMRI test of spatiotopic representation is that such a representation may be masked by the more rapidly changing retinotopic representations, especially given the kind of theoretical analyses presented here which suggest a preponderance of retinotopic representations, such as retinotopic boundary, surface, surface contour, and eye command representations, that are nested among a smaller number of spatiotopic representations, such as binocular boundary and attentional shroud representations (Figures 2–4). Finer neurophysiological methods will likely be needed to sort out these retinotopic and spatiotopic differences, as they have begun to in past research.

Some behavioral experiments report a brief retinotopic facilitation (priming) effect followed by a sustained spatiotopic IOR effect (Posner and Petersen, 1990). The kind of stimuli in these experiments include attending to events in a given visual position, covert shifts in attention or orienting to a new position upon cuing, visual search (Posner and Cohen, 1984; Posner, 1988), as well as letter and word matching (Posner, 1978). Some behavioral measures for such data are collated from reaction times to efficiently respond to activities in the cued location (Posner, 1988), enhanced scalp electrical activity (Mangoun and Hillyard, 1987), higher discharge rates of neurons in several areas of the monkey brain (Mountcastle, 1978; Wurtz et al., 1980; Petersen et al., 1987), spared abilities of patients with lesions and monkeys with chemical lesions in different areas of the brain (Posner and Cohen, 1984; Posner et al., 1984; Posner, 1988), and how each area and hemispheric differences affects the ability to engage in attention, orient or remain alert to a target (Gazzaniga, 1970; Sergent, 1982; Robertson and Delis, 1986).

The brief facilitation was due to the activation of retinotopic units representing the stimulus, in which case, the selection of a response occurs more quickly than when not expecting a target to occur or when targets occur without warning. This selection of a response, though, is based upon a lower quality of information about the classification of the target stimulus, resulting in an increase in error rate to respond to the stimulus. This increase in errors, while not affecting the build-up of information in the retinotopic system, affects the rate at which attention can respond to the stimulus leading to a sustained spatiotopic IOR. 3D ARTSCAN mechanisms are compatible with such data, since the retinotopic representations are used to build spatiotopic representations, and shroud IOR mechanisms are computed in spatiotopic coordinates.

Various experiments find persistent spatiotopic facilitation along with short-term retinotopic facilitation in certain task conditions (Golomb et al., 2008, 2010a,b). Thus, contextual relevance of tasks may play a role in whether object locations are coded in retinotopic or head-centered/spatiotopic coordinates systems. For example, in Golomb et al. (2008), the manipulation of the Stimulus Onset Asynchrony of the probe stimulus enabled the tracking of when the transition between retinotopic and spatiotopic coordinates occurs. In one of the experiments to sustain a stable spatiotopic representation, immediately after a saccade, attention is primarily maintained at the previously relevant retinotopic coordinates of the cue. However, after 100–200 ms, the task-relevant spatiotopic coordinates start to dominate and the retinotopic facilitation decays. On the other hand, when the experiment was modified to make the retinotopic location the task-relevant location and the spatiotopic location task-irrelevant, the retinotopic location was facilitated over the entire delay period of 75–600 ms probed. This kind of manipulation gives insight into the temporal dynamics of spatial attention and the mechanisms by which attention is maintained across saccades.

### 6.8. Remapping of border-ownership in V2 and attentive enhancement in V1

The electrophysiological experiments of O'Herron and von der Heydt (2013) on border-ownership neurons in visual cortical area V2 of monkeys showed that there is remapping of border-ownership signals when the retinal image moves either due to saccades or object movements. A border-ownership neuron responds to borders with differing firing rates depending on whether the border is owned by a figure on one side or the other. The difference in firing rates to the two conditions is defined as the border-ownership signal. An ambiguous edge was used as a probe in both cases. In the saccade paradigm, the edge of a figure (square) is presented outside the cell receptive field (RF) in the first phase. This is substituted by the ambiguous edge in the second phase. In the third phase, a saccade is induced to move the RF into the ambiguous edge. The V2 neuron did not respond during the first two phases, but responded when the saccade brought the RF onto the edge. The difference in the response was related to neither the direction of the saccade nor the location of the figure relative to the RF, but to the initial border-ownership. The border-ownership defined by the figure edge was inherited by the ambiguous edge and transferred across cortex at the time of saccade. In the object movement paradigm, the displays used in the first two phases were the same as for the saccades paradigm. In the third phase, instead of moving the fixation point (as was done in the saccade condition), the figure edge along with the object were moved to have the edge land in the RF of the neuron. The results were similar to those of the saccade experiment in terms of the amplitudes of the transferred signals. The response onset and rise of the border-ownership signal in the object movement were more abrupt and aligned to the edge movement. For the saccade condition, they were aligned with the movement of the fixation point and the response onset varied with saccade latency. This remapping of border-ownership was observed in both the paradigms at the V2 population level as well.

Border-ownership modulation of neurons in area V2 is akin to the remapping often observed in neurons in areas controlling visual attention and planning of eye movements, in which a stimulus activates a neuron whose RF has not yet seen the stimulus (e.g., Duhamel et al., 1992), showing that remapping may occur in low-level visual areas as well.

The FACADE and 3D LAMINART models have simulated a number of figure-ground percepts using model neural mechanisms in V2. These percepts include Bregman-Kanizsa figure-ground separation and various lightness percepts, including the Munker-White, Benary cross, and checkerboard percepts (Kelly and Grossberg, 2000), percepts of Kanizsa stratification, transparency, and 3D neon color spreading (Grossberg and Yazdanbakhsh, 2005), and bistable percepts, including their modulation by attention, such as the percept of a Necker cube (Grossberg and Swaminathan, 2004) and binocular rivalry (Grossberg et al., 2008). Because these models can be consistently added to the pre-processing levels in 3D LAMINART, they can be explained in this model in a manner consistent with the figure-ground remapping results.

A study involving a curve tracing task, with multi-unit activity recorded from monkey visual cortical area V1, established remapping of response modulation for attentive enhancement (Khayat et al., 2004). In this work, the monkeys performed a curve tracing task, and had to make two successive saccades along a single curve to which they were attending, while ignoring another curve. Response enhancement for the neurons representing the selected curve was observed. After the first saccade, there was enhancement in the response of the neurons representing the curve in the new retinal locations. Response modulation appeared in neurons that had not been activated initially, and the attentive enhancement was remapped, or transferred across cortex. This response modulation to attentive enhancement in V1 is strikingly similar to the predictive remapping often observed in neurons in LIP and other areas that control visual attention and planning of predictive eye movements and requires the selective attention of one stimulus over the other for response modulation.

The two studies summarized above appear to differ in the role of attention in remapping, but are complementary and can be integrated within the 3D ARTSCAN model. To achieve such remapping, both the systems need to compute the displacement vector of the shift. In predictive remapping, this displacement information is provided by the outflow command of the eye movement centers, which update gain fields that drive the remapping. The similarity of the results for saccades or object movement in the border-ownership in V2, and the response modulation in V1 to attentive enhancement, are consistent with the remapping via gain fields, that is used in the 3D ARTSCAN model, and lend further support to the FAÇADE theory claim that figure-ground mechanisms for boundary formation, and thus for their remapping, can occur at early stages of visual cortex. Despite frequent saccades or displacement on the retina, early remapping is essential to maintain assignment of local features to an external object. Such congruity serves as a crucial step toward building object invariance, and enabling the integration of details of the object into a coherent percept.

### Conflict of interest statement

The authors declare that the research was conducted in the absence of any commercial or financial relationships that could be construed as a potential conflict of interest.
